# RNA-Seq analysis of European sea bass (*Dicentrarchus labrax* L.) infected with nodavirus reveals powerful modulation of the stress response

**DOI:** 10.1186/s13567-020-00784-y

**Published:** 2020-05-12

**Authors:** Raquel Lama, Patricia Pereiro, Valentina Valenzuela-Muñoz, Cristian Gallardo-Escárate, Lluis Tort, Antonio Figueras, Beatriz Novoa

**Affiliations:** 1 Institute of Marine Research (IIM), National Research Council (CSIC), Eduardo Cabello, 6, 36208 Vigo, Spain; 2 Laboratory of Biotechnology and Aquatic Genomics, Interdisciplinary Center for Aquaculture Research (INCAR), University of Concepción, P.O. Box 160, Concepción, Chile; 3 Department of Cell Biology, Physiology and Immunology, Autonomous University of Barcelona, 08193 Barcelona, Spain

## Abstract

Nodavirus, or nervous necrosis virus (NNV), is the causative agent of viral encephalopathy and retinopathy (VER), a severe disease affecting numerous fish species worldwide. European sea bass, a cultured species of great economic importance, is highly susceptible to the disease. To better understand the response of this organism to NNV, we conducted RNA-Seq analysis of the brain and head kidney from experimentally infected and uninfected sea bass juveniles at 24 and 72 hours post-infection (hpi). Contrary to what was expected, we observed modest modulation of immune-related genes in the brain, the target organ of this virus, and some of these genes were even downregulated. However, genes involved in the stress response showed extremely high modulation. Accordingly, the genes encoding the enzymes implicated in the synthesis of cortisol were almost the only overexpressed genes in the head kidney at 24 hpi. This stress response was attenuated after 72 h in both tissues, and a progressive immune response against the virus was mounted. Moreover, experiments were conducted to determine how stress activation could impact NNV replication. Our results show the complex interplay between viral activity, the stress reaction and the immune response.

## Introduction

European sea bass (*Dicentrarchus labrax* L.) is a very valuable fish species in Mediterranean countries, and it is currently one of the main cultured fish species in Europe [1]. However, different infectious diseases can affect its production and cause important economic impacts in the aquaculture industry. One of the most significant diseases affecting *D. labrax* is viral encephalopathy and retinopathy (VER), which is characterized by severe damage to nervous tissues [2]. The causative agent of this disease is nervous necrosis virus (NNV), or nodavirus, belonging to family *Nodaviridae,* genus *Betanodavirus*. NNV is a naked, icosahedral, single-stranded, positive-sense RNA virus [2]. The *Betanodavirus* genus is composed of 4 genotypes that infect different animal species [3], among which European sea bass seems to be mainly affected by the red-spotted grouper nervous necrosis virus (RGNNV) genotype [2, 4]. Due to its virulence and rapid spreading, it is associated with high mortality rates, reaching 100% in many cases, and although this disease mostly affects juveniles, it has also been detected in adult animals [2, 4]. Because of its neurotropic nature, NNV mainly affects the brain and retina of infected fish. When the nervous system of an individual is affected, it manifests very specific symptoms, such as erratic swimming in descending circles, which can cause curvature of the dorsal spine, and other less specific symptoms (exophthalmia, bloated abdomen and anorexia).

Bioinformatic tools allow the in-depth study of the interactions between an infected organism and its pathogen. Several studies have used high-throughput RNA sequencing (RNA-Seq) to understand the effects of nodavirus via the transcriptome profiling of in vitro-infected cells. Such investigations have been performed in grouper kidney cells (GK cell line) [5], Asian sea bass epithelial cells (SB cell line) [6], European sea bass leukocytes [7], striped snakehead fish cells (SSN-1 cell line) [8] and European sea bass brain cells (DLB-1 cell line) [9]. The in vivo effect of NNV has also been analysed by RNA-Seq in the brain of sevenband grouper [10], pooled brain/eye and head kidney samples from Senegalese sole [11], the brain of Malabar grouper [12], and the liver, spleen and kidney of *Epinephelus moara* [13]. However, the in vivo response of European sea bass remains almost completely unexplored, and only a small number of publications have reported the modulation or involvement of immune factors in different tissues of *D. labrax* infected with NNV [14–22]. Therefore, the aim of this work was to analyse the complete transcriptome response of European sea bass to nodavirus infection. As the materials for this study, we selected the main target organ of this virus, the brain, as well as the head kidney because it plays crucial roles in the organization of both immune and stress responses. Interestingly, the induction of immune genes was practically undetectable, but we observed strong modulation of genes related to the hypothalamic-pituitary-interrenal (HPI) axis. Although numerous publications reported the effect of a variety of stressors in the susceptibility to diseases and their impact on different immune parameters [23], the stress response induced by pathogens remains practically unexplored in fish. However, pathogens are considered as an important environmental biotic stressor for plants and animals. This is the first time that RNA-Seq analysis has shown an interaction between neuroendocrine pathways and the immune system through the HPI axis during nodavirus infection.

## Materials and methods

### Fish and virus

Healthy juvenile specimens of European sea bass (*Dicentrarchus labrax* L.) were obtained from the facilities of the Estación de Ciencias Mariñas de Toralla (ECIMAT, Universidad de Vigo, Spain) (average body weight of ~70 g) or from the Naturix Cantabria hatchery (Cantabria, Spain) (average body weight of ~10 or ~50 g). Prior to the experiments, the fish were acclimatized to the laboratory conditions for 2 weeks; they were maintained in 500-litre fibreglass tanks with a re-circulating saline-water system (total salinity approximately 35 g/L) under a light–dark cycle of 12:12 h at 20–22 °C and were fed daily with a commercial diet. The animals were euthanized via an MS-222 overdose. All the experimental procedures were reviewed and approved by the CSIC National Committee on Bioethics under approval number ES360570202001/17/FUN.01/INM06/BNG.

The viral strain 475-9/99, belonging to the RGNNV genotype, was provided by the Institute Zooprofilattico delle Venize (Italy) after isolation from diseased sea bass [24]. The snakehead-fish cell line SSN-1 (ECACC 96082808) was grown at 25 °C in Leibovitz’s L15-medium (Gibco, Carlsbad, CA, USA) supplemented with 10% heat-inactivated foetal bovine serum (FBS) (Gibco), 1% l-glutamine (Gibco) and a 1% penicillin/streptomycin solution (Invitrogen). The virus was propagated in the SSN-1 cell line in the medium described above supplemented with 2% FBS, with incubation at 25 °C until the cytopathic effect was extensive. The supernatants were harvested and centrifuged to eliminate cell debris. Clarified supernatants were used in all infections. The viral titre, expressed as TCID_50_/mL (tissue culture infectious dose infecting 50% of inoculated cultures), was determined in 96-well plates according to the endpoint titration procedure [25].

### Fish infection and sampling for RNA-Seq

Sedated specimens of European sea bass (~70 g) were intramuscularly (i.m.) injected with 100 μL of SSN-1 culture medium (control) or with culture medium containing NNV at 10^6^ TCID_50_/mL (infected). A total of 9 fish per condition were sampled at 24 and 72 hpi. The same quantity of tissue from 3 animals was pooled, performing 3 biological replicates (3 fish/replicate) per tissue at each sampling point. The brain and head kidney were harvested under RNase-free conditions and stored at −80 °C until RNA isolation. In parallel, the effect of the viral challenge in the survival was analysed to confirm the virulence of the NNV in those animals sampled for the RNA-Seq analysis. For this, ten animals were inoculated with NNV or SSN-1 culture medium as mentioned above, and mortality was recorded during the next 2 weeks.

### RNA isolation and high-throughput transcriptome sequencing

RNA extraction was performed with the Maxwell 16 LEV simply RNA tissue kit (Promega). The RNA concentration and purity were measured with a spectrophotometer (ND-1000; Nanodrop Technologies Inc., Wilmington, DE, USA), and RNA integrity was analysed in an Agilent 2100 Bioanalyzer (Agilent Technologies Inc., Santa Clara, CA, USA) according to the manufacturer’s instructions. All the samples showed an RNA Integrity Number (RIN) over 8.0 and were therefore used for Illumina library preparation. Double-stranded cDNA libraries were constructed using the TruSeq RNA Sample Preparation Kit v2 (Illumina, San Diego, CA, USA), and sequencing was performed using Illumina HiSeq 4000 technology at Macrogen Inc., Korea (Seoul, Republic of Korea). The read sequences were deposited in the Sequence Read Archive (SRA) under accession number PRJNA589774.

### Raw data cleaning, de novo assembly and gene annotation

CLC Genomics Workbench v. 11.0.2 (CLC Bio, Aarhus, Denmark) was used for filtration and assembly and to perform the RNA-Seq and statistical analyses. Prior to assembly, the raw data from each sample were trimmed to remove adapter sequences and low-quality reads (quality score limit 0.05). All the high-quality reads were de novo assembled in a unique file using default parameters (mismatch cost = 2, insert cost = 3, minimum contig length = 200 bp, and similarity = 0.8). The contigs yielded by this assembly were annotated with the Blast2GO program against the UniProtKB/SwissProt database with a cutoff E-value of 1E−03.

### RNA-Seq analysis

The transcriptome database generated for European sea bass was used as a reference for the RNA-Seq analyses (mismatches = 2, length fraction = 0.8, similarity fraction = 0.8). Expression levels were calculated as transcripts per million (TPM) values. To determine statistically significant differences, a proportion-based statistical analysis was conducted using Baggerly’s test and adjusting *p*-values by the false discovery rate (FDR) correction. Contigs showing a fold change > 2 in the absolute value in relation to the control group and an FDR < 0.05 were selected as differentially expressed genes (DEGs). Heat maps were constructed by plotting the log_2_ values of the normalized TPM values and were hierarchically clustered by estimating Manhattan distances with an average linkage criterion. Finally, functional annotation was performed at the online site DAVID 6.8 to conduct Gene Ontology (GO) enrichment and KEGG pathway analyses using the UniProtIDs of our DEG lists. For the GO and KEGG analyses, a *p* < 0.05 was employed.

### NNV detection and RNA-Seq validation by quantitative PCR (qPCR)

cDNA synthesis from the sequenced samples was performed with the NZY First-Strand CDNA Synthesis Kit (NZYtech, Lisbon, Portugal) using 0.1 µg of total RNA. The qPCR assays were performed using specific primers, and their efficiencies were previously tested according to the method described by Pfaffl [26]. Individual qPCR assays were conducted in a 25-μL reaction volume including 12.5  μL of SYBR GREEN PCR Master Mix (Applied Biosystems, Foster City, CA, USA), 10.5  μL of ultrapure water (Sigma-Aldrich, St. Louis, MO, USA), 0.5  μL of each specific primer (10  μM) and 1  μL of cDNA template. All reactions were performed in technical triplicates in a 7300 Real-Time PCR System thermocycler (Applied Biosystems) with an initial denaturation step (95 °C, 10 min), followed by 40 cycles of a denaturation step (95 °C, 15  s) and a singly hybridization-elongation step (60 °C, 30  s). Relative gene expression was calculated via the Pfaffl method [26] and using *18S ribosomal RNA* (*18S*) as a reference gene. Fold units were calculated by dividing the normalized expression values of the different samples by the normalized expression values obtained in the controls.

To detect viral replication, a 203-bp PCR product from the NNV coat protein gene (RNA2) was chosen for its specificity and the absence of PCR artefacts or primer dimers [27].

Twelve genes that were significantly modulated in the brain or head kidney were chosen for the validation of the RNA-Seq results. The sequences of the primers used for NNV detection and RNA-Seq validation are listed in Additional file 1. Additionally, a biological validation was conducted in an independent experiment. For this, ten sea bass (~50 g) were i.m. injected with 100 μL of SSN-1 culture medium (control) or with culture medium containing NNV at 10^6^ TCID_50_/mL (infected). Five individual brain samples were taken at 24 and 72 hpi. Three genes that were significantly modulated in the RNA-Seq data were selected for qPCR analysis.

### Cortisol implants and nodavirus challenge

A cortisol implant was prepared by dissolving cortisol (Sigma-Aldrich) in coconut oil (Sigma-Aldrich) at a final concentration of 50 µg cortisol/g body weight [28]. Juvenile sea bass (~10 g) were anaesthetized, intraperinoteally (i.p.) injected with 100 µL of the cortisol implant or the vehicle alone (coconut oil), and them i.m. injected with 100 µL of an NNV suspension at 2 × 10^4^ TCID_50_/mL (infected) or the culture medium alone (control). The mortality of each treatment (two replicates of 20 fish each) was recorded.

In parallel, forty fish were divided into 4 tanks (10 fish/tank) and each group was inoculated as mentioned above for the survival experiment. Brain samples were collected at 24 and 72 hpi from each group (*n* = 5 individuals). The total brain was homogenized and half of the tissue was used for RNA isolation and the other half for protein extraction. RNA was used to analyse by qPCR the replication of the virus and the expression of two genes that were significantly modulated according to the RNA-Seq results: the immune gene encoding IgM (*ighm*), and the gene encoding the stress hormone prolactin (*prl*).

The remaining tissue was used for IgM detection via an enzyme-linked immunosorbent assay (ELISA). Frozen samples were homogenized in 400 µL of buffer containing 150 mM NaCl, 10 mM Tris–HCl, 1 mM EGTA, 1 mM EDTA (pH 7.4), 1% Triton X-100, 0.5% NP40-IGEPAL, 1 × Halt phosphatase inhibitor cocktail (Sigma-Aldrich) and 1× protease inhibitor cocktail (Sigma-Aldrich). The tubes were kept on ice throughout the process to prevent protein denaturation. The homogenates were centrifuged at 1000 *g* for 15 min at 4 °C, and the supernatants were centrifuged again at 20 000 *g* for 30 min at 4 °C. The resulting supernatants were recovered and stored at −80 °C. The concentration of protein in each sample was determined using a NanoDrop ND-1000 spectrophotometer. Protein extracts were diluted in 20 mM Tris–HCl (pH 4) at a proportion of 1 µg per 50 µL, and this volume was dispensed into each well of a 96-well flat-bottom high-binding plate (Costar, Cambridge, MA, USA), which was then incubated overnight at 37 °C. A 100-µL volume of an 8% non-fat dry milk solution in each well was used for blocking at room temperature (RT) for 4 h. Then, the wells were washed three times with distilled water. For the detection of the protein, 50 µL of a solution of the anti-IgM monoclonal antibody (Aquatic Diagnostics Ltd., Stirling, Scotland, UK) diluted 1:33 in ELISA buffer (0.5% BSA, 0.01% Tween 20, 0.005% phenol red and 10% PBS in distilled water; pH 7) was added to the wells, followed by incubation for 2 h at RT and then washing three times with distilled water. The same volume of ELISA buffer alone at the same concentration was used as a negative control. A 50-µL volume per well of a goat anti-mouse IgG antibody labelled with horseradish peroxidase (HRP) (Sigma-Aldrich) diluted 1:500 in ELISA buffer was applied as the secondary antibody for the detection of the specific IgM-primary antibody interaction, followed by incubation for 45 min at RT. After three washes, 100 µL of the 1-Step Ultra TMB-ELISA solution (Thermo Scientific, Waltham, MA, USA) was added to each well. The reaction was stopped with 2 N H_2_SO_4_, and the optical density was measured at 450 nm with a spectrophotometer (Labsystems iEMS Reader MF). The intensity of the control signal was subtracted from the intensity of the signal obtained with the anti-IgM antibody. These values were directly proportional to the amount of IgM protein present in each well.

### Respiratory burst activity determined by chemiluminescence assays

To verify the capacity of the infected cells to produce reactive oxygen species (ROS), we analysed the burst activity of kidney leukocytes during in vitro and in vivo infections. For the in vitro assay, the head kidneys were collected under sterile conditions in Leibovitz’s medium 1 × (L15) and subjected to forced passage through a 100 µm nylon mesh. The obtained head kidney cell suspensions were layered over a 51% Percoll (GE Healthcare, Chicago, IL, USA) density gradient and centrifuged at 400 *g* for 30 min at 4 °C. After centrifugation, the band of leucocytes above the Percoll-medium interface was collected with a Pasteur pipette, washed twice with L15 and centrifuged at 400 *g* for 10 min at 4 °C. The cell pellet was resuspended in L15 supplemented as the SSN-1 medium. The cells were counted and adjusted to a concentration of 10^6^ cells/mL. The cell suspensions were distributed in 96-well opaque white plates with a flat bottom and low evaporation lid (Falcon), and infected (or not) with NNV (10^4^ TCID_50_/mL) for 1, 24, 48 and 72 h before ROS production measurement. For the in vivo assay, animals (~10 g) were i.m. infected (or not) with NNV (10^4^ TCID_50_/mL) and at 1 and 5 days post-infection (dpi), head kidney samples were taken, and leukocytes were collected as described above, then plated in opaque white plates.

The emission of relative luminescence units (RLU) was determined after the stimulation of the cells with phorbol myristate acetate (PMA, Sigma-Aldrich) and amplified by the addition of 5-amino-2,3-dihydro-1,4-phthalazinedione (Luminol, Sigma-Aldrich). A stock solution of 0.1 M luminol was prepared in dimethyl sulfoxide (DMSO, Sigma-Aldrich) just before use. Luminol was diluted in phosphate-buffered saline (PBS, Gibco) to obtain a working solution with a final concentration of 10–4 M. The PMA stock (1 mg/mL in DMSO) was also diluted in the luminol working solution to obtain a final concentration of 1 µg/mL. The working solutions of luminol either alone or in combination with PMA were added to the wells, and the generation of chemiluminescence was measured after 5 min in a luminometer (Fluoroskan Ascent, Labsystems, Vantaa, Finland). Four individual biological replicates and triplicate wells were assayed.

### Inactivation of NNV using hydrogen peroxide

Hydrogen peroxide (H_2_O_2_) has been used to study the effect of ROS produced during viral infection on nodavirus replication. A 30% stock solution of H_2_O_2_ (Perhydrol; Merck, Darmstadt, Germany) was sterilized by filtration through a 0.22 µm-pore filter and kept in a dark sealed container. The viral suspension (10^4^ TCID_50_/mL) was treated with the H_2_O_2_ solution at a final concentration of 3% and incubated for 5 or 24 h at 4 °C. To stop the reaction and remove residual H_2_O_2_, the viral suspensions were treated twice with 12.5 U/mL of catalase from bovine liver (Sigma-Aldrich) for 10 min at room temperature. Viral suspensions without H_2_O_2_ treatment were incubated under the same conditions and served as a control for the infection, and aliquots of H_2_O_2_-catalase treated medium were also included as a control for the treatment. Then, SSN-1 cells seeded in 24-well plates were inoculated with these suspensions (4 wells per condition) and incubated at 25 °C for 72 h. After this period, viral replication was analysed by qPCR.

### Intracellular calcium measurement with FLUO-4 AM

For in vitro experiments, SSN-1 cells were distributed in 24-well plates, infected with NNV (10^4^ TCID_50_/mL) and incubated at 25 °C for 24, 72 and 96 h. Noninfected controls were also included. For in vivo experiments, fish were i.m. injected with NNV (10^4^ TCID_50_/mL) or culture medium, and at 1 and 5 dpi, primary cell cultures of the brain were obtained (4 biological replicates per condition and sampling point). The entire brain was sampled; the meninges were completely removed by dissection; and the brain was finally collected in Hanks’ Balanced Salt Solution without calcium chloride or magnesium sulphate (HBSS, Sigma-Aldrich), washed three times and subjected to forced passage through an 80 µm nylon mesh. The obtained cell suspension was centrifuged at 300 *g* for 10 min at 4 °C. The cell pellet was resuspended in Leibovitz’s medium 1 × (L15) supplemented as the SSN-1 medium. The brain cells were counted and adjusted to a concentration of 10^6^ cells/mL.

For both experiments cells were washed with PBS and loaded with 10 µL of FLUO-4 AM (Sigma-Aldrich) diluted in L15 without phenol red (Sigma-Aldrich) to avoid interfering with the fluorescence of the probe. After an incubation period of 2.5 h at 25 °C, the cells were washed, followed by 45 min of de-esterification. The cells were then counted and resuspended at a concentration of 2 × 10^5^ cells/mL in L15 without phenol red. The cell suspensions were distributed in black 96-well plates (Cliniplate-Thermo Scientific, Waltham, MA, USA) to measure changes in fluorescence with an excitation wavelength of 485 nm and emission wavelength of 538 nm (Fluoroskan Ascent, Labsystems, Vantaa, Finland). Each sample was measured in triplicate.

### Cell treatment with calcium channel inhibitors and calcium chelators

We used different inhibitors of cellular calcium channels and calcium chelators to study the effect of cytoplasmic calcium on NNV replication and ROS production. As inhibitors of calcium channels, we used thapsigargin (Tg; Sigma-Aldrich), which causes calcium release from the endoplasmic reticulum; carboxyamidotriazole (CAI; Sigma-Aldrich), which is an inhibitor of non-voltage-dependent calcium entry and inhibits mitochondrial calcium import; nitrendipine (Sigma-Aldrich), a calcium entry blocker shown to inhibit the movement of calcium through L-type calcium channels; and verapamil hydrochloride (Sigma-Aldrich), which inhibits calcium movement across cell membranes, both inward and outward. As calcium chelators, we selected the intracellular calcium chelator BAPTA-AM (Sigma-Aldrich) and the extracellular calcium chelator EGTA (Sigma-Aldrich). The final concentrations used for the experiments were as follows: Tg (5 µM), CAI (0.5 µM), nitrendipine (0.5 µM), BAPTA-AM (50 µM), and EGTA (60 µM).

To analyse the effect of the changes in calcium homeostasis on NNV replication, SSN-1 cells were distributed in 24-well plates and pretreated for 2 h with the calcium chelators and pharmacological inhibitors, then washed twice with PBS and infected with NNV (10^4^ TCID_50_/mL) for 48 h at 25 °C. Viral replication was analysed by qPCR in 5 biological replicates per treatment.

To determine ROS production in the presence of the calcium chelators, primary cultures of head kidney leukocytes were pretreated for 2 h with the calcium chelators BAPTA-AM and EGTA, washed twice with PBS, and inoculated with NNV (infected) or control medium (control) for 1 h. ROS production was measured as described above with 8 biological replicates and 3 technical replicates.

### Statistical analysis

Kaplan–Meier survival curves were analysed with a log-rank (Mantel-Cox) test. The correlation between the RNA-Seq and qPCR data was analysed by using Pearson’s correlation coefficient. For the remaining experiments, the results were represented graphically as the mean + standard error of the mean (SEM), and significant differences were determined using Student’s *t*-test. For the comparisons among different sampling points the significant differences were stablished using one-way ANOVA (poshoc Tukey’s multiple comparison test). Statistically significant differences were indicated as ***/^###^(0.0001 < *p* < 0.001), **/^##^(0.001 < *p* < 0.01) or */^#^(0.01 < *p* < 0.05).

## Results

### Effects of NNV challenge in juvenile sea bass

**Figure 1:**
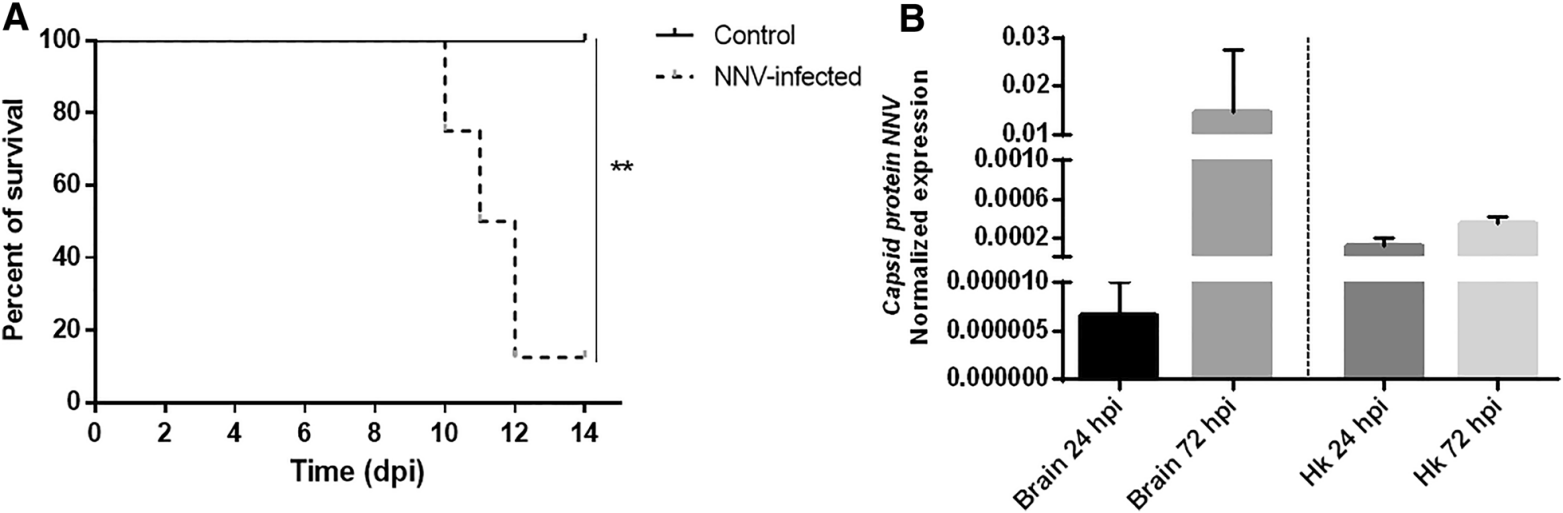
**Effects of NNV intramuscular infection in juvenile *D. labrax.*A** Kaplan–Meier survival of NNV-infected and uninfected fish. **B** Replication of NNV at 24 and 72 hpi in brain and head kidney samples. The level of viral replication was measured by qPCR amplification of the gene encoding the NNV capsid.

European sea bass challenged with NNV began to manifest clinical signs of infection at 8 dpi; these signs mainly consisted of erratic swimming behaviours, such as spiral or whirling swimming, belly-up floating, and laying down at rest (Additional file 2). After 12 days, the survival rate of the infected individuals was only 16.5% (Figure 1A). In parallel to this mortality assay, we collected brain and head kidney samples from infected and uninfected animals at 24 and 72 hpi, which were used for RNA-Seq analyses. NNV replication was confirmed by qPCR in these samples and, although statistically significant differences were not observed between 24 and 72 hpi, the viral replication tended to increase with time in the brain, whereas the uninfected control samples were negative for the detection of the virus (Figure 1B). As expected, higher replication levels were detected in the brain, which is the target tissue of NNV, after 72 h.

### Transcriptome modulation in European sea bass after NNV infection

A summary of the average reads per sample, assembly data and contig annotation is provided in Table 1. After adapter trimming and quality filtering of the raw data, an average of 28 044 852.6 high-quality reads per sample were obtained, with a mean length of 101 bp. These reads were de novo assembled and yielded a total of 347 317 contigs. Among these contigs, 10.2% were successfully annotated against the UniProtKB/Swiss-Prot database with an E-value cutoff of 1E–03.

**Table 1:** Summary of Illumina sequencing, assembly and annotation.

Description	Value
Raw data	
Reads per sample	28 044 852.60
Average lenght (bp)	101
Number of samples	24
De novo assembly	
Number of contigs	347 317
Average length (bp)	723
N75 (bp)	453
N50 (bp)	1088
N25 (bp)	2851
UniprotKB/swiss-prot blast	
Successfully annotated contigs (%)	10.2

**Figure 2:**
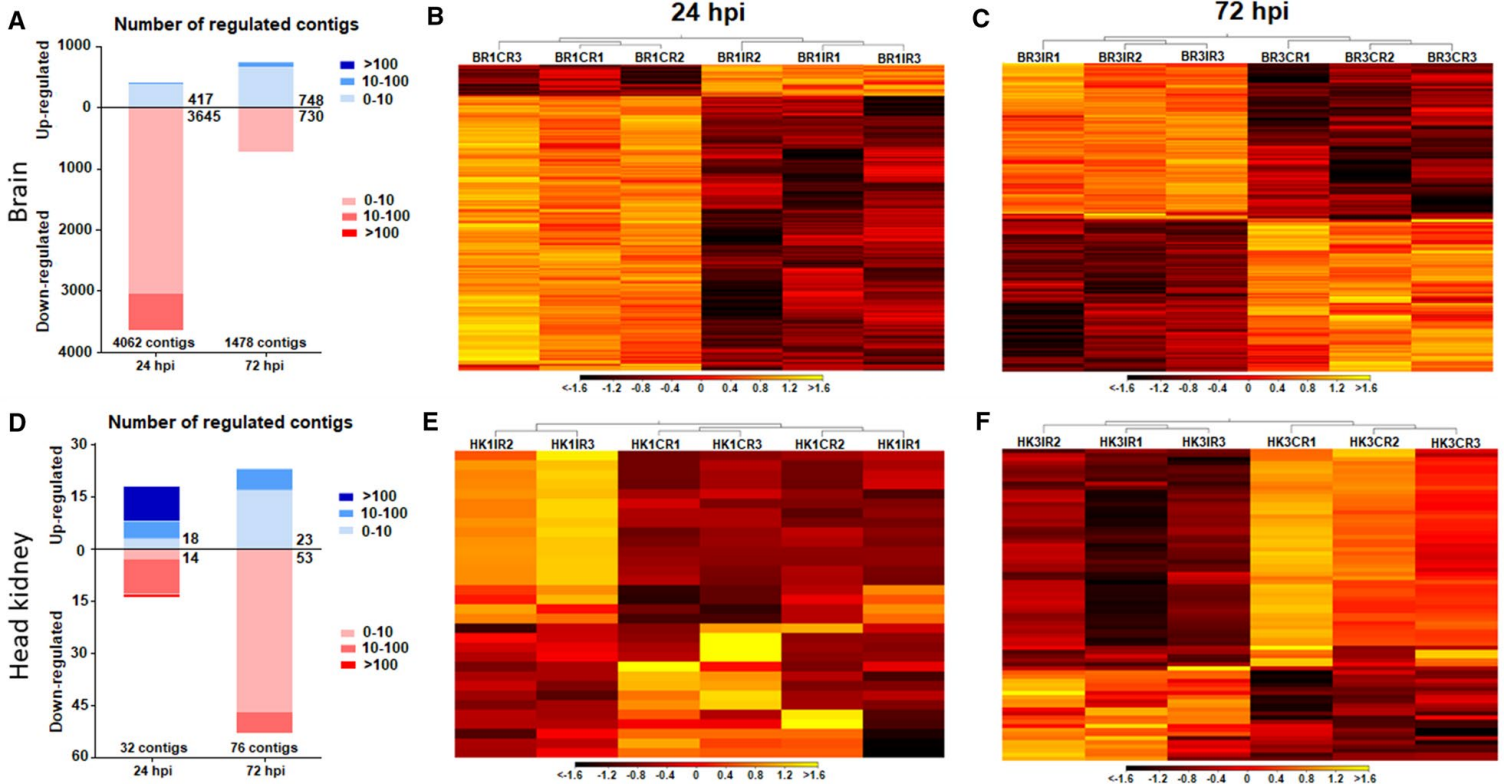
**Differentially expressed genes in the brain and head kidney after NNV challenge.** Stacked column charts reflect the number and intensity of the DEGs identified in the brain **A** and head kidney **D** at 24 and 72 hpi (FC > |2|; FDR < 0.05). Heat maps representing the log_2_-transformed TPM expression values of the DEGs at 24 and 72 hpi for the brain **B**, **C** and head kidney **E**, **F**. Colours represent transcript expression ranging from black (less expressed) to yellow (more expressed).

RNA-Seq analyses were conducted to evaluate transcriptome modulation in the brain and head kidney during infection with nodavirus. Using the obtained data, DEGs between the infected and uninfected fish were identified according to an FC > |2| and FDR < 0.05. As expected, due to the neurotropic nature of the virus, a greater number of DEGs were registered in the brain, which showed 4062 transcripts that were differentially regulated at 24 hpi and 1478 at 72 hpi (Figure 2A; Additional file 3). On the other hand, only 32 and 76 DEGs were registered in the head kidney at 24 and 72 hpi, respectively (Figure 2D; Additional file 4). Both the stacked column chart (Figure 2A) and the corresponding heat map (Figure 2B) showed that most of the transcripts that were significantly modulated in the brain at 24 hpi were inhibited by the viral challenge; however, the response seemed to be more equilibrated at 72 hpi (Figures 2A–C), which was also observed in the head kidney (Figures 2D–F).

**Figure 3:**
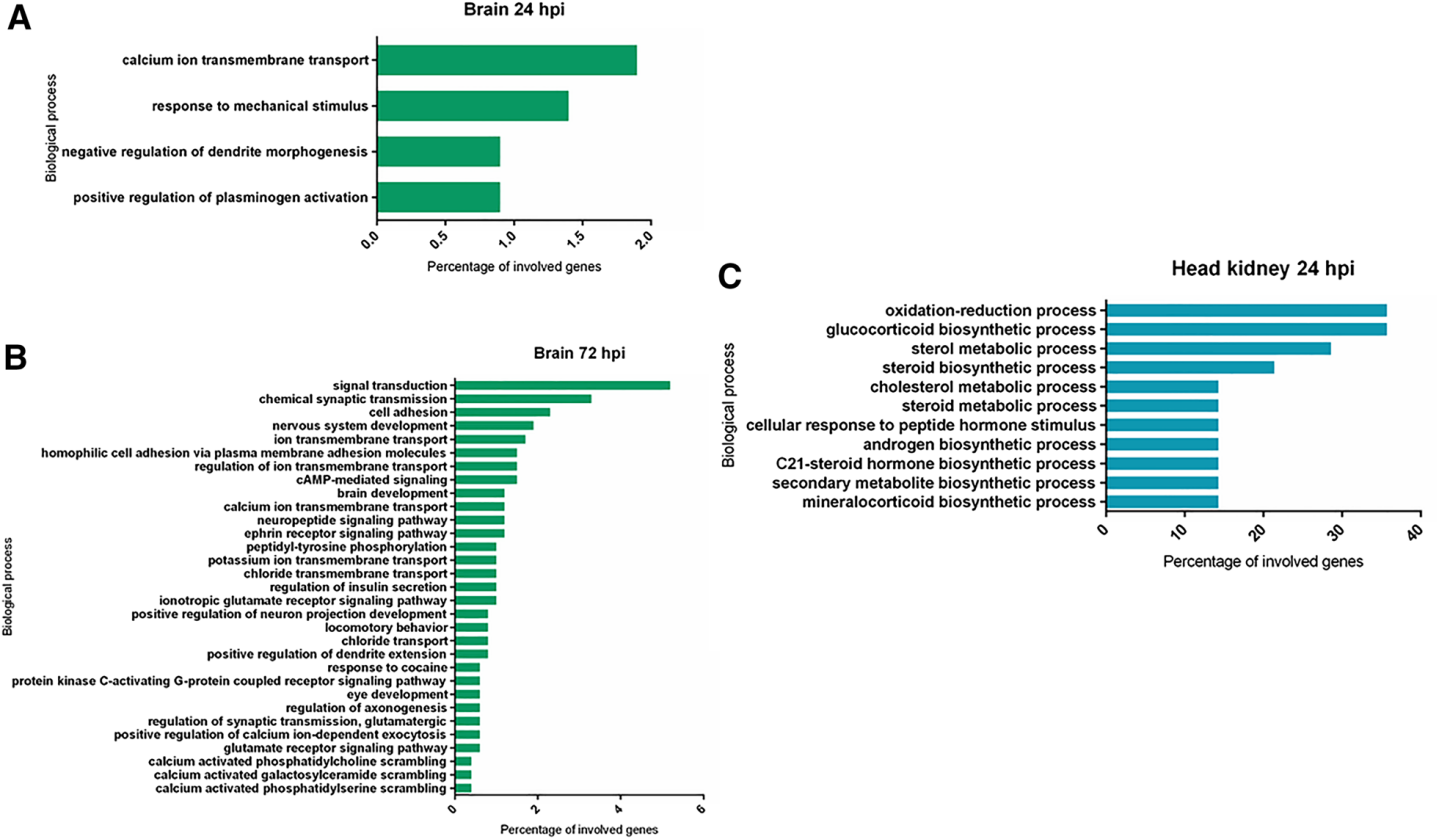
**GO biological processes enriched in the brain at 24.A** and 72 hpi **B** and in the head kidney at 24 hpi **C**. No enriched terms were observed for the head kidney at 72 hpi.

GO enrichment analyses were carried out to explore the biological processes that were enriched during infection. In the brain, only four biological processes were significantly enriched at 24 hpi: “calcium ion transmembrane transport”, “response to mechanical stimulus”, “negative regulation of dendrite morphogenesis” and “positive regulation of plasminogen activation” (Figure 3A). However, although the number of DEGs in the brain was lower at 72 hpi, the number of enriched terms was 31 in this case, and many of them were related to ion transport and processes regulated by calcium or mediated by different neurotransmitters or their receptors (Figure 3B). In the case of the head kidney, enriched biological processes were only obtained at 24 hpi, with the terms “oxidation–reduction process”, “glucocorticoid biosynthetic process” and “sterol metabolic process” being the most enriched (Figure 3C).

### Modulation of immune-related genes after NNV infection

Whereas almost no typical immune genes seemed to be significantly modulated by viral challenge in the head kidney at the analysed sampling points, some of these genes were differentially expressed in the brain (Table 2). At 24 hpi, only 10 immune-related contigs were modulated by the infection, including the overexpression of a c-type lectin, the chemotaxin *lect2* and the *nktr* receptor of natural killer cells. Interestingly, three relevant immune genes were downregulated at this sampling point: the *immunoglobulin mu chain C region secreted form* (*ighm*), the *stimulator of interferon genes protein* (*sting*) and the *NLR family CARD domain containing 3* (*nlrc3*). After 72 h, the number of immune genes that were modulated by the infection was also low, with the only overexpressed genes being *pentraxin 4* (*ptx4*), *viperin* or *radical S*-*adenosyl methionine domain*-*containing protein 2* (*rsad*), *complement component c3* (*co3*) and *complement c1q tumour necrosis factor*-*related protein 1* (*c1qt1*) (Table 2).

**Table 2:** Immune-related genes significantly modulated in the brain after NNV challenge.

Gene Name	24 hpi	72 hpi
Leukocyte cell-derived chemotaxin 2 (LECT2)	*3.54*	***−4.89***
C-type lectin (TETN)	*3.85*	
T-cell leukemia homeobox protein 3 (TLX3)	*2.47*	
Platelet-derived growth factor receptor (PGFR)	*2.35*	*2.15*
NK-tumor recognition protein (NKTR)	*2.14*	
Eomesodermin (EOMES)	*2.06*	
Probable ATP-dependent RNA helicase DDX17 (DDX17)	*2.01*	
Ig mu chain C region secreted form (IGHM)	***−2.91***	
Stimulator of interferon genes protein (STING)	***−4.13***	
NLR family CARD domain containing 3 (NLRC3)	***−8.7***	
Pentraxin-4 (PTX4)		*13.24*
Radical S-adenosyl methionine domain-containing protein 2 (RSAD2)		*6.25*
Complement component C3 (CO3)		*5.66*
Complement C1q tumor necrosis factor-related protein 1 (C1QT1)		*2.15*
B-cell lymphoma/leukemia 11A (BC11A)		***−2.09***
Semaphorin-4C (SEM4C)		***−2.12***
Nitric oxide synthase, brain (NOS1)		***−2.39***
E3 ubiquitin-protein ligase TRIM39 (TRIM39)		***−2.66***
Platelet glycoprotein V (GPV)		***−3.52***
Leukocyte cell-derived chemotaxin 1 (CNMD)		***−4.88***

### Nodavirus induces alterations in the HPI axis

#### HPI axis modulation in the brain

**Figure 4:**
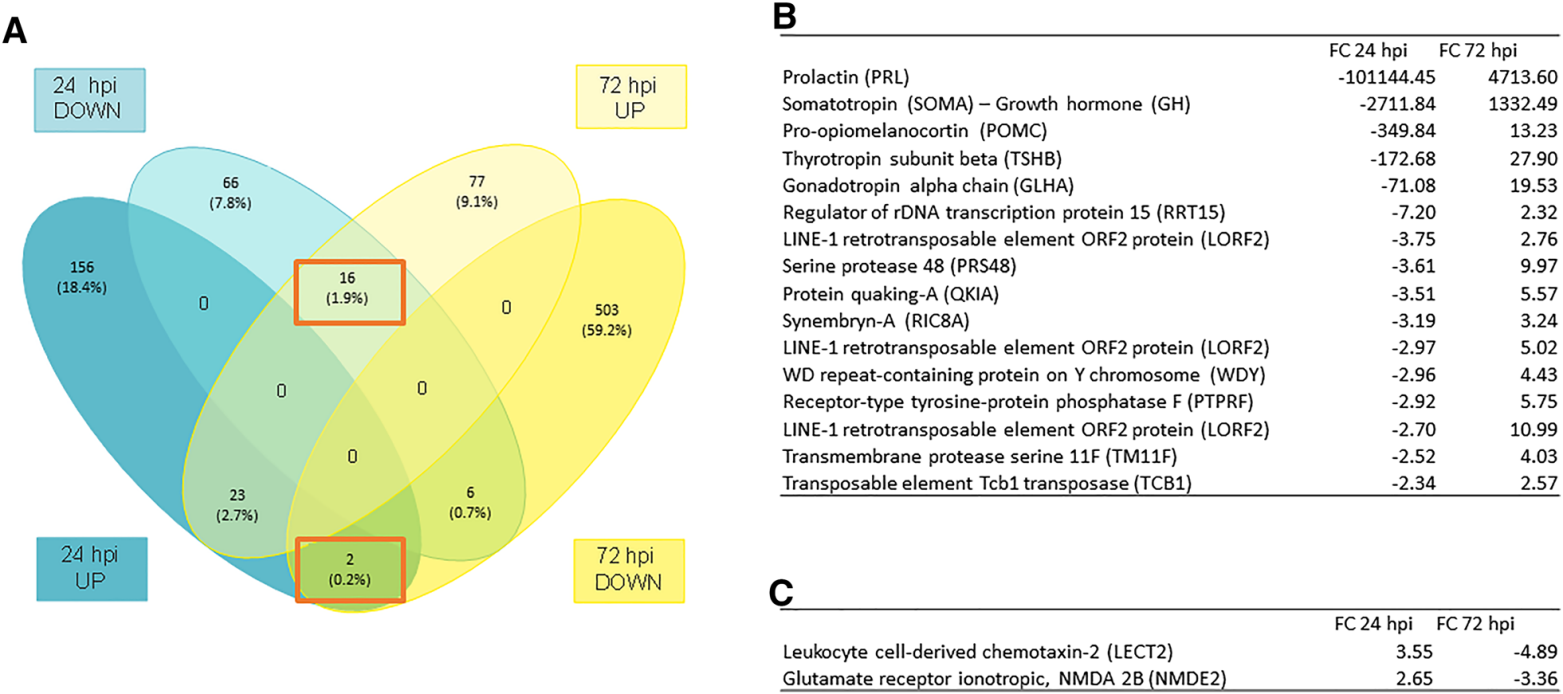
**Venn diagram of the up- and downregulated DEGs identified in the brain at 24 and 72 hpi. **Some contigs showed a reversal of their modulation from upregulated to downregulated, or vice versa, between 24 and 72 hpi.

As reflected in the top 25 modulated contigs identified in the brain (Table 3), numerous genes encoding hormones involved in the HPI axis were greatly modulated after NNV infection. These genes corresponded to the pituitary hormones *prolactin* (*prl*), *somatolactin* (*sl*), *somatotropin* (*soma*) or *growth hormone* (*gh*), *gonadotropin* (*glha*), *thyrotropin* (*tsh*) and *pro*-*opiomelanocortin* (*pomc*), the last of which encodes the precursor protein of adrenocorticotropic hormone (ACTH). Interestingly, a Venn diagram of the DEGs identified in the brain at 24 and 72 hpi revealed that among the 47 DEGs that were commonly modulated at both sampling points, there were 16 contigs that switched from being downregulated at 24 hpi to upregulated at 72 hpi (Figure 4A). These genes included those encoding the pituitary hormones *prl*, *gh*, *glh*, *tsh* and *pomc* (Figure 4B). Therefore, from 24 to 72 hpi, there was a complete shift in the expression of these HPI-related genes. On the other hand, only 2 common DEGs between 24 and 72 hpi showed a change from being upregulated at 24 to downregulated at 72 hpi, which corresponded to *leukocyte cell*-*derived chemotaxin*-*2* (*lect2*) and the *glutamate receptor ionotropic nmde2* gene, encoding the neurotransmitter receptor Nmde2 (Figure 4C).

**Table 3:** Top 25 up- and down-regulated DEGs in the brain at 24 and 72 hpi.

Up		Down	
Blast2GO UniProt/SwissProt description	FC	Blast2GO UniProt/SwissProt description	FC
24 hpi			
Hydroxytryptamine receptor 3E (5HT3E5)	51.82	Prolactin (PRL)	−101144.45
Bile acid-CoA:amino acid N-acyltransferase (BAAT)	13.07	Somatolactin (SL)	−13670.06
Actin-related protein 2/3 complex subunit 1A(ARC1A)	12.76	Somatotropin (SOMA)–growht hormone (GH)	−2711.84
Collagen alpha-1(X) chain (COAA1)	10.87	Gonadotropin subunit beta-2 (GTHB2)	−1896.99
B2 bradykinin receptor (BKRB2)	7.36	O-acyltransferase like protein (OACYL)	−377.22
Deoxyribonuclease-1 (DNAS1)	6.95	Pro-opiomelanocortin (POMC)	−349.84
Deoxyribonuclease-1 (DNAS1)	6.48	Serotransferrin-1 (STF)	−240.04
Alkaline phosphatase, tissue-nonspecific isozyme (PPBT)	6.20	Thyrotropin subunit beta (TSHB)	−172.68
Proline-rich basic protein 1 (PROB1)	4.78	Gonadotropin alpha chain (GLHA)	−71.08
Probable cysteine–tRNA ligase, mitochondrial (SYCM)	4.68	Transmembrane protein 130 (TM130)	−27.42
MAGUK p55 subfamily member 4 (MPP4)	4.34	LINE-1 type transposase domain-containing protein 1 (LITD1)	−18.39
Acidic repeat-containing protein (ACRC)	4.20	Pituitary-specific positive transcription factor 1 (PIT1)	−16.88
Gag-Pol polyprotein (POL)	4.14	Troponin T, fast skeletal muscle isoforms (TNNT3)	−16.50
MICAL-like protein 2 (MILK2)	4.12	Transcription factor COE1 (COE1)	−16.04
Leucine-rich repeat and coiled-coil domain-containing protein 1 (LRCC1)	4.07	Methylmalonyl-CoA mutase, mitochondrial (MUTA)	−15.94
Tetranectin (TETN)	3.86	Gonadotropin subunit beta-1 (GTHB1)	−12.59
ATP-binding cassette sub-family A member 1 (ABCA1)	3.81	LINE-1 reverse transcriptase homolog (LIN1)	−10.49
Oxysterol-binding protein-related protein 11 (OSB11)	3.78	Pituitary homeobox 3 (PITX3)	−10.15
DNA replication complex GINS protein PSF3 (PSF3)	3.58	Myosin regulatory light chain 2, skeletal muscle isoform type 2 (MLRS)	−9.10
Filamin-C (FLNC)	3.57	NLR family CARD domain containing 3 (NLRC3)	−8.70
Leukocyte cell-derived chemotaxin-2 (LECT2)	3.55	LINE-1 retrotransposable element ORF2 protein (LORF2)	−8.66
Deoxynucleoside triphosphate triphosphohydrolase SAMHD1 (SAMH1)	3.50	UPF0577 protein KIAA1324 (K1324)	−8.58
Protein largen (LARGN)	3.41	NADH dehydrogenase (NDUFA1)	−7.48
High affinity choline transporter 1 (SC5A7)	3.37	Regulator of rDNA transcription protein 15 (RRT15)	−7.20
Cathepsin D (CATD)	3.36	Fructose-bisphosphate aldolase A (ALDOA)	−6.90
72 hpi			
Prolactin (PRL)	4713.60	Myosin regulatory light chain 2, skeletal muscle isoform type 2 (MLRS)	−47.94
Somatotropin (SOMA)	1332.49	Collagen alpha-1(II) chain (CO2A1)	−34.45
Thyrotropin subunit beta (TSHB)	27.90	Transposon Ty3-I Gag-Pol polyprotein (YI31B)	−30.32
Probable cysteine–tRNA ligase, mitochondrial (SYCM)	25.81	Fructose-bisphosphate aldolase A (ALDOA)	−19.98
Gonadotropin alpha chain (GLHA)	19.53	Troponin T, fast skeletal muscle isoforms (TNNT3)	−18.43
Pentraxin-4 (PTX4)	13.24	Sodium channel protein type 4 subunit alpha B (SC4AB)	−14.00
Pro-opiomelanocortin (POMC)	13.23	Sarcoplasmic/endoplasmic reticulum calcium ATPase 1 (AT2A1)	−13.68
LINE-1 retrotransposable element ORF2 protein (LORF2)	10.99	Creatine kinase M-type (KCRM)	−12.46
Serine protease 48 (PRS48)	9.97	Actin, alpha skeletal muscle 2 (ACT2)	−11.73
Probable E3 ubiquitin-protein ligase HERC4 (HERC4)	8.55	Keratin, type I cytoskeletal 17 (K1C17)	−11.31
Sacsin (SACS)	7.07	Parvalbumin alpha (PRVA)	−10.45
C-X-C motif chemokine 9 (CXCL9)	7.07	Collagen alpha-1(II) chain (CO2A1)	−9.10
Radical S-adenosyl methionine domain-containing protein 2 (RSAD2)	6.63	Keratin, type I cytoskeletal 13 (K1C13)	−8.06
Sacsin (SACS)	6.37	DNA damage-inducible transcript 4-like protein (DDT4L)	−5.60
Receptor-transporting protein 3 (RTP3)	5.81	Receptor-type tyrosine-protein phosphatase F (PTPRF)	−5.58
Receptor-type tyrosine-protein phosphatase F (PTPRF)	5.75	Metabotropic glutamate receptor 8 (GRM8)	−5.54
Complement C3 (CO3)	5.66	Alpha-1B adrenergic receptor (ADA1B)	−5.46
Protein quaking-A (QKIA)	5.57	Sodium channel protein type 4 subunit alpha B (SC4AB)	−5.30
Inter-alpha-trypsin inhibitor heavy chain H2 (ITIH2)	5.03	Amiloride-sensitive amine oxidase (AOC1)	−5.15
LINE-1 retrotransposable element ORF2 protein (LORF2)	5.02	DNA damage-inducible transcript 4-like protein (DDT4L)	−5.03
Dynein heavy chain 8, axonemal (DYH8)	4.99	Bifunctional heparan sulfate N-deacetylase/N-sulfotransferase 3 (NDST3)	−5.02
Retrovirus-related Pol polyprotein from transposon 412 (POL4)	4.73	Leukocyte cell-derived chemotaxin-2 (LECT2)	−4.89
Inter-alpha-trypsin inhibitor heavy chain H5 (ITIH5)	4.50	Leukocyte cell-derived chemotaxin 1 (CNMD)	−4.89
WD repeat-containing protein on Y chromosome (WDY)	4.43	Feline leukemia virus subgroup C receptor-related protein 2 (FLVC2)	−4.83
Filamin-C (FLNC)	4.37	Sulfotransferase family cytosolic 2B member 1 (ST2B1)	−4.79

Neurotransmitters and their receptors are also involved in the activation and/or deactivation of the HPI axis. Indeed, we observed that a large number and various types of neurotransmitter receptors were modulated during infection with NNV (Table 4). In general, although with some exceptions, we observed overexpression of some neurotransmitter receptors at 24 hpi but downregulation at 72 hpi (Table 4). Those neurotransmitter receptors that were overexpressed at 24 hpi corresponded to serotonin and glutamate receptors, whereas at 72 h, serotonin and glutamate receptors were mainly inhibited, as were acetylcholine and adrenergic receptors. Although some GABA receptors were slightly inhibited after 72 h, *gbrb1* was overexpressed (FC = 4.26), and it was the most differentially modulated GABA receptor. Among all the modulated receptors, the highest fold change was observed for the serotonin receptor *5*-*hydroxytryptamine receptor 3E* (*5ht3e*) at 24 hpi, which was overexpressed in infected animals by 52-fold compared to control fish.

**Table 4:** Genes encoding neurotransmitter receptors significantly modulated in the brain after NNV infection.

Type of neurotransmitter receptor	Receptor or related protein	24 hpi	72 hpi
Serotonin receptors	5-hydroxytryptamine receptor 3E (5HT3E)	*51.82*	
	5-hydroxytryptamine receptor 5A (5HT5A)		***−3.19***
	5-hydroxytryptamine receptor 1F (5HT1F)		***−2.74***
Glutamate receptors	N-methyl-d-aspartate (NMDA) NMDA 1		***−2.35***
	N-methyl-d-aspartate (NMDA) NMDA 2A		***−2.77***
	N-methyl-d-aspartate (NMDA) NMDA 2B	*2.65*	***−3.36***
	N-methyl-d-aspartate (NMDA) NMDA 2C		*2.22*
	N-methyl-d-aspartate (NMDA) NMDA 2D		***−2.38***
	α-amino-3-hydroxy-5-methyl-4-isoazolepropionic acid (AMPA) AMPA1 (GluR-1)		***−2.32***
	α-amino-3-hydroxy-5-methyl-4-isoazolepropionic acid (AMPA) AMPA2 (GluR-2)	*2.05*	
	α-amino-3-hydroxy-5-methyl-4-isoazolepropionic acid (AMPA) AMPA3 (GluR-3)		***−2.68***
	α-amino-3-hydroxy-5-methyl-4-isoazolepropionic acid (AMPA) AMPA4 (GluR-4)		***−2,06***
	2-carboxy-3-carboxymethyl-4-isopropenylpyrrolidine (kainate) 5 (GluK5)		***−2,04***
	Metabotropic glutamate receptor 1 (mGluR1)	*2.12*	
	Metabotropic glutamate receptor 4 (mGluR4)		***−2,14***
	Metabotropic glutamate receptor 5 (mGluR5)		***−3.28***
	Metabotropic glutamate receptor 8 (mGluR8)		***−5.54***
	Vesicular glutamate transporter 1 (VGluT1)	*2.03*	
	Vesicular glutamate transporter 2.1 (VGL2A)		***−2.24***
	AMPA receptor-interacting protein GRIP1		*2.10*
	Glutamate receptor-interacting protein 2 (GRIP2)	*2.01*	2.01 &−2.05
	Kainate-binding protein. Glutamate receptor U1 (GLRK)	*2.06*	
	Sodium-dependent glutamate/aspartate transporter 1 (EAA1, GLAST-1)	*2.51*	***−2.39***
GABA receptors	GABA(A) receptor subunit alpha-2 (GBRA2)		***−2.85***
	GABA(A) receptor subunit alpha-5 (GBRA5)		***−2.63***
	GABA(A) receptor subunit beta-1 (GBRB1)		*4.26*
	GABA(A) receptor subunit beta-3 (GBRB3)		***−3.07***
	GABA(A) receptor subunit beta-4 (GBRB4)		***−2.41***
	GABA(A) receptor subunit pi (GBRP)		***−2.43***
Acetylcholine receptors	Muscarinic acetylcholine receptor M2 (ACM2)		***−2.14***
	Neuronal acetylcholine receptor subunit alpha-3 (ACHA3)		***−3.14***
	Neuronal acetylcholine receptor subunit alpha-3 (ACHA3)		***−2.70***
	Neuronal acetylcholine receptor subunit alpha-7 (ACHA7)		***−3.24***
Adrenergic receptors	Alpha-1B adrenergic receptor (ADA1B)		***−5.46***
	Alpha-2B adrenergic receptor (ADA2B)		***−2.01***
	Alpha-2Db adrenergic receptor (AA2DB)		***−2.70***

**Figure 5:**
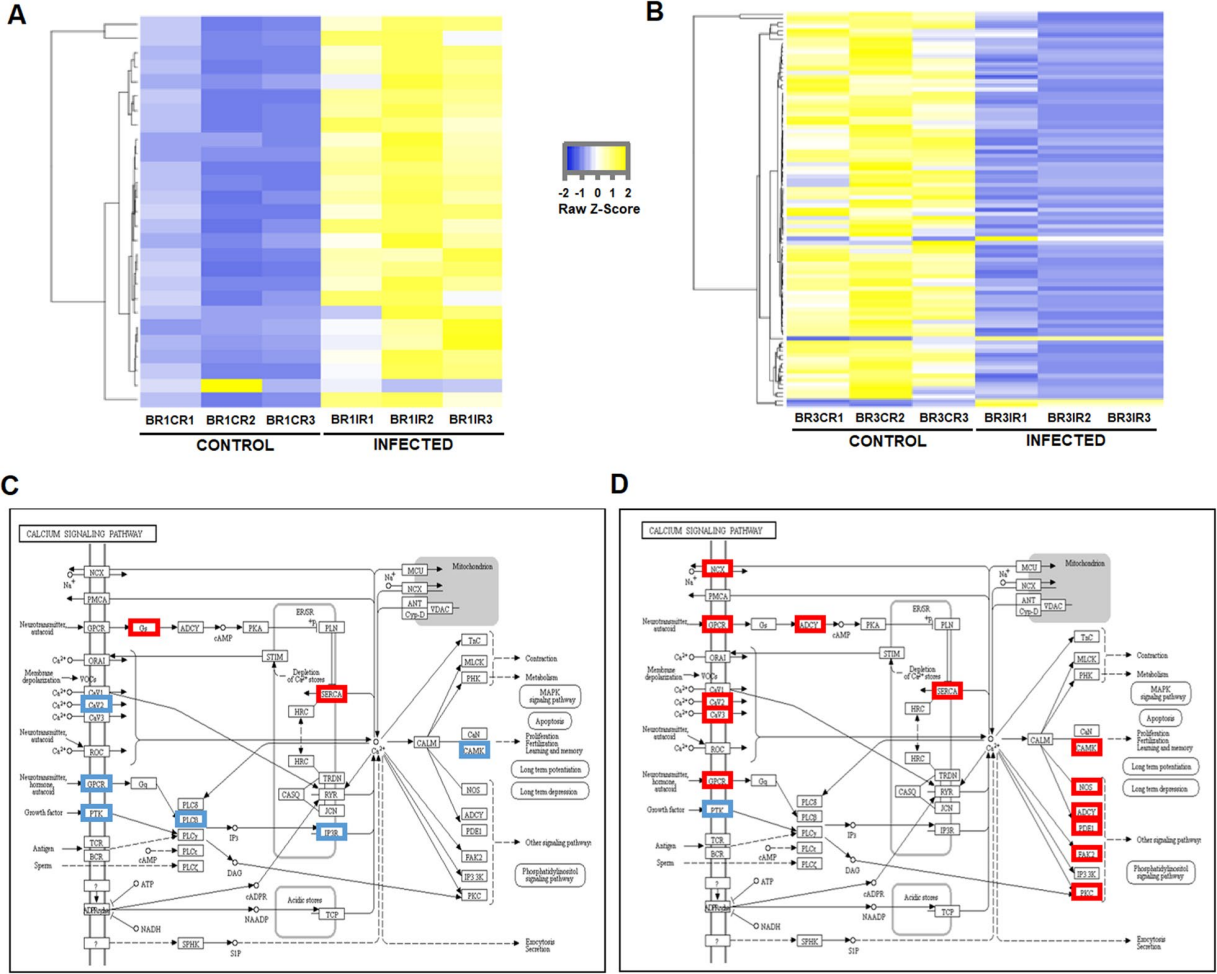
**Modulation of DEGs related to calcium transport and homeostasis in the brain at 24 and 72 hpi.** Heat maps representing the log_2_-transformed TPM expression values of the calcium-related contigs at 24 **A** and 72 hpi **B**. Colours represent transcript expression ranging from blue (less expressed) to yellow (more expressed). Representation of the modulation of the calcium signalling KEGG pathway components according to the RNA-Seq results at 24 **C** and 72 hpi **D**.

Among the enriched GO terms associated with the brain during NNV infection, we also found high representation of terms related to calcium homeostasis (Figures 3A, B). Excessive stimulation by neurotransmitters can cause excitotoxicity by increasing the massive influx of calcium ions into cells [29]. Therefore, a similar pattern to that observed for the neurotransmitters could be expected for the genes encoding calcium transporters. When the DEGs included among the enriched biological processes related to calcium were represented in a heat map, we generally observed the overexpression of the calcium-related genes at 24 hpi (Figure 5A), but most of these genes were downregulated at 72 hpi (Figure 5B). Based on the KEGG pathway analysis of calcium signalling, we identified the main cellular calcium regulators affected by infection with NNV at both sampling points (Figures 5C, D). As expected based on previous observations, at 24 hpi the changes in gene expression seemed to favour higher levels of calcium in the cytoplasm (overexpression of several calcium import channels), whereas at 72 hpi, the opposite response was observed (downregulation of calcium importers).

Therefore, NNV infection induced strong modulation of genes related to the HPI axis in the brain by affecting the expression of the genes encoding neurotransmitter receptors, calcium transporters and pituitary hormones. However, the tendencies were mainly opposite between the two sampling points.

#### HPI axis modulation in the head kidney

Adrenocorticotropic hormone (ACTH) stimulates the production of cortisol, the main stress hormone, by the interrenal cells of the head kidney. When we analysed the effect of NNV in head kidney samples, we found that there were only 17 annotated DEGs at 24 hpi, and most of the upregulated genes encoded enzymes involved in the final steps of the steroid hormone biosynthesis pathway, specifically related to the transformation of cholesterol into cortisol (Table 5; Additional file 5): *cytochrome p450 11b*, *mitochondrial* (*cyp11b1*), *steroidogenic acute regulatory protein*, *mitochondrial* (*star*), *steroid 21*-*hydroxylase* (*cyp21a*), *cytochrome p450 17a1* (*cyp17a1*), *3 beta*-*hydroxysteroid dehydrogenase* (*3bhsd*) and *cholesterol side*-*chain cleavage enzyme*, *mitochondrial* (*cyp11a1*). None of these genes were significantly modulated after 72 h.

**Table 5:** Top 25 up- and downregulated DEGs in the head kidney at 24 and 72 hpi.

Up		Down	
Blast2GO UniProt/SwissProt description	FC	Blast2GO UniProt/SwissProt description	FC
24 hpi			
Cytochrome P450 11B, mitochondrial (CP11B)	7007.72	Transcription cofactor vestigial-like protein 4 (VGLL4)	−88.03
Steroidogenic acute regulatory protein, mitochondrial (STAR)	1423.20	Keratin, type I cytoskeletal 13 (K1C13)	−71.73
Cytochrome P450 11B, mitochondrial (CP11B)	453.32	NLR family CARD domain-containing protein 3 (NLRC3)	−64.71
Steroid 21-hydroxylase (CP21A)	349.77	Keratin, type II cytoskeletal 8 (K2C8)	−60.16
Steroid 21-hydroxylase (CP21A)	326.02	Translocase of chloroplast 34 homolog, chloroplastic (TOC34)	−15.80
Steroid 17-alpha-hydroxylase/17,20 lyase (CP17A)	307.93	Carbonic anhydrase 1 (CAH1)	−5.18
Steroid 17-alpha-hydroxylase/17,20 lyase (CP17A)	95.58		
3 beta-hydroxysteroid dehydrogenase/Delta 5– > 4-isomerase type 1 (3BHSD)	61.79		
Nuclear receptor subfamily 5 group A member 2 (NR5A2)	43.00		
Chemokine-like receptor 1 (CML1)	17.97		
Cholesterol side-chain cleavage enzyme, mitochondrial (CP11A)	4.37		
72 hpi			
Acidic mammalian chitinase (CHIA)	32.07	Contactin-associated protein-like 5 (CNTP5)	−39.47
Transposon Ty3-I Gag-Pol polyprotein (YI31B)	10.35	Elongation factor 1-gamma (EF1G)	−32.60
Myosin-binding protein C, fast-type (MYPC2)	6.51	Phosphoserine aminotransferase (PSAT)	−6.24
Fructose-bisphosphate aldolase A (ALDOA)	5.12	Lanosterol 14-alpha demethylase (CP51A)	−6.08
Neuronal acetylcholine receptor subunit beta-4 (ACHB4)	2.84	D-3-phosphoglycerate dehydrogenase (3-PGDH)	−5.03
Glyceraldehyde-3-phosphate dehydrogenase (G3P)	2.75	Calreticulin (CALR)	−4.42
Rieske domain-containing protein (RFESD)	2.08	Protein FAM111A (F111A)	−4.16
		Calreticulin (CALR)	−3.74
		Mitochondrial glutamate carrier 1 (GHC1)	−3.43
		Arachidonate 12-lipoxygenase, 12R-type (LX12B)	−3.20
		Ubiquitin carboxyl-terminal hydrolase isozyme L3 (UCHL3)	−3.06
		Fibronectin (FINC)	−3.05
		Argininosuccinate synthase (ASSY)	−3.04
		Quinone oxidoreductase PIG3 (QORX)	−2.88
		Calreticulin (CALR)	−2.76
		Stromal cell-derived factor 2-like protein 1 (SDF2L)	−2.75
		Endoplasmic reticulum chaperone BiP (BIP)	−2.71
		Inositol-3-phosphate synthase 1-A (INO1A)	−2.69
		Protein disulfide-isomerase A6 (PDIA6)	−2.63
		Spermidine synthase (SPEE)	−2.62
		Nuclear mitotic apparatus protein 1 (NUMA1)	−2.49
		Protein disulfide-isomerase A4 (PDIA4)	−2.47
		Rac GTPase-activating protein 1 (RGAP1)	−2.45
		L-lactate dehydrogenase A chain (LDHA)	−2.40
		Histone chaperone asf1b-B (AS1BB)	−2.36

### Effect of cortisol on defence against NNV

**Figure 6:**
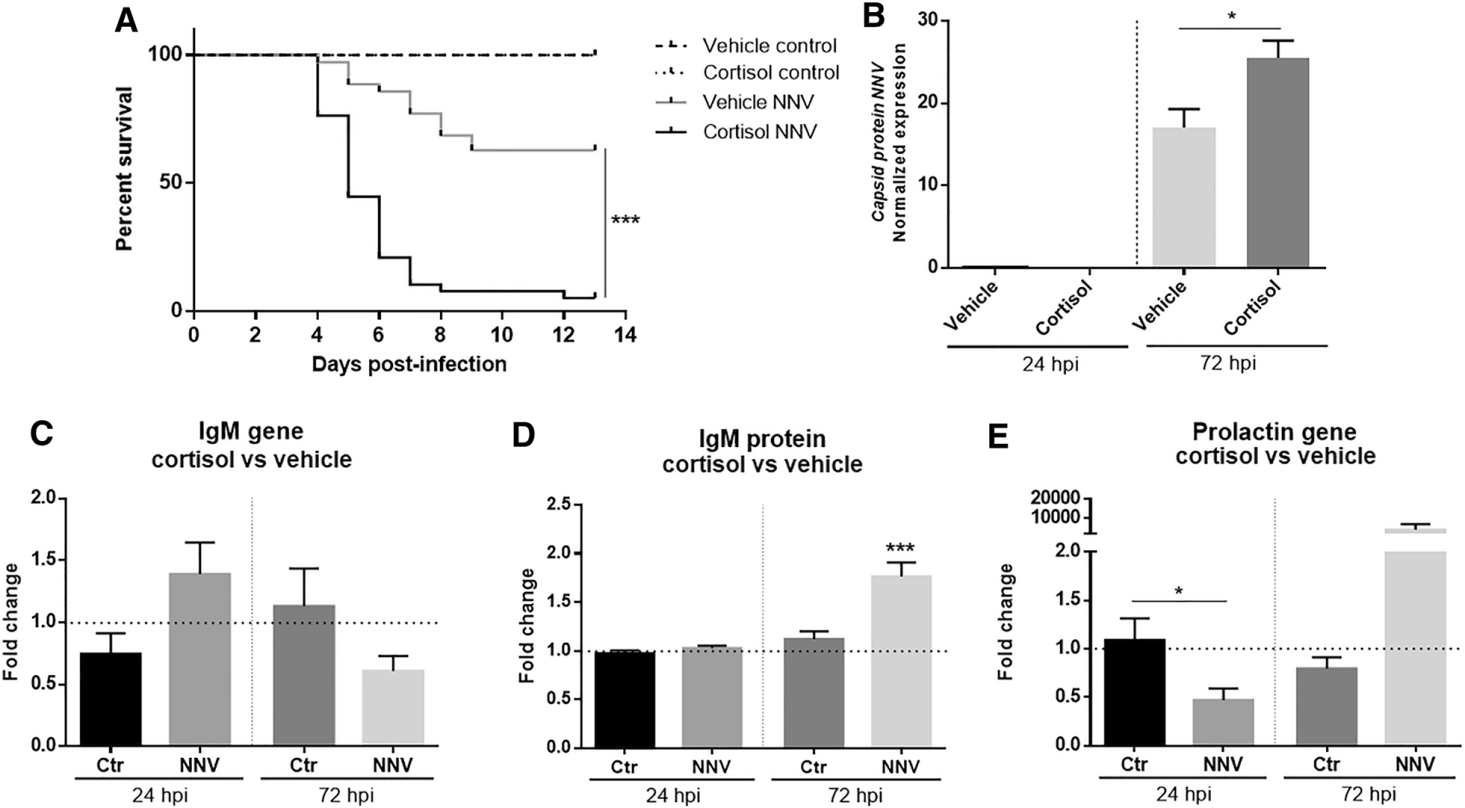
**Cortisol effects during NNV infection.A** Kaplan–Meier survival curve representing the higher NNV susceptibility of sea bass carrying intraperitoneal slow-release cortisol implants compared to those inoculated with the vehicle alone. **B** NNV replication in the brain of sea bass carrying the cortisol implants or the vehicle alone (*n* = 5 individual samples). Viral replication was detected by qPCR amplification of the gene encoding the NNV capsid. **C** Expression of the *ighm* gene in NNV-infected or uninfected fish in the absence or presence of cortisol overload. The data are represented as fold change expression level in those animals carrying cortisol implants compared to those inoculated with the vehicle. **D** Detection of the IgM protein by ELISA in NNV-infected or uninfected fish carrying cortisol implants or the vehicle alone (*n* = 5 individual samples). The data are represented as fold change of protein abundance in those animals carrying cortisol implants compared to those inoculated with the vehicle. **E** Expression of the *prl* gene in NNV-infected or uninfected fish in the absence or presence of cortisol overload. The data are represented as fold change expression level in those animals carrying cortisol implants compared to those inoculated with the vehicle.

The modest modulation of immune genes contrasted with the broad, intense changes in the expression of the genes involved in the HPI axis. The individuals carrying the cortisol implant showed a lower survival rate (5.3%) compared to those inoculated with the vehicle alone (62.9%) (Figure 6A), which was associated with a higher NNV level detected by qPCR at 72 hpi (Figure 6B).

Interestingly, cortisol did not significantly affect the expression of the *ighm* gene in the absence or presence of NNV infection (Figure 6C). Surprisingly, when the IgM protein content was analysed, higher levels of IgM were detected at 72 hpi in the animals infected with NNV and carrying cortisol implants compared to those infected but injected with the vehicle (Figure 6D). Therefore, although our results showed an increase in sea bass susceptibility to NNV with cortisol overload, it seems that this lower resistance was not mediated by IgM synthesis suppression. We also observed higher expression of *prl* associated with the combination of cortisol overload and NNV infection after 72 hpi compared to those animals inoculated with the vehicle and infected with NNV, although due to the high deviation among the samples, differences were not statistically significant (Figure 6E).

### ROS production modulation during nodavirus infection

**Figure 7:**
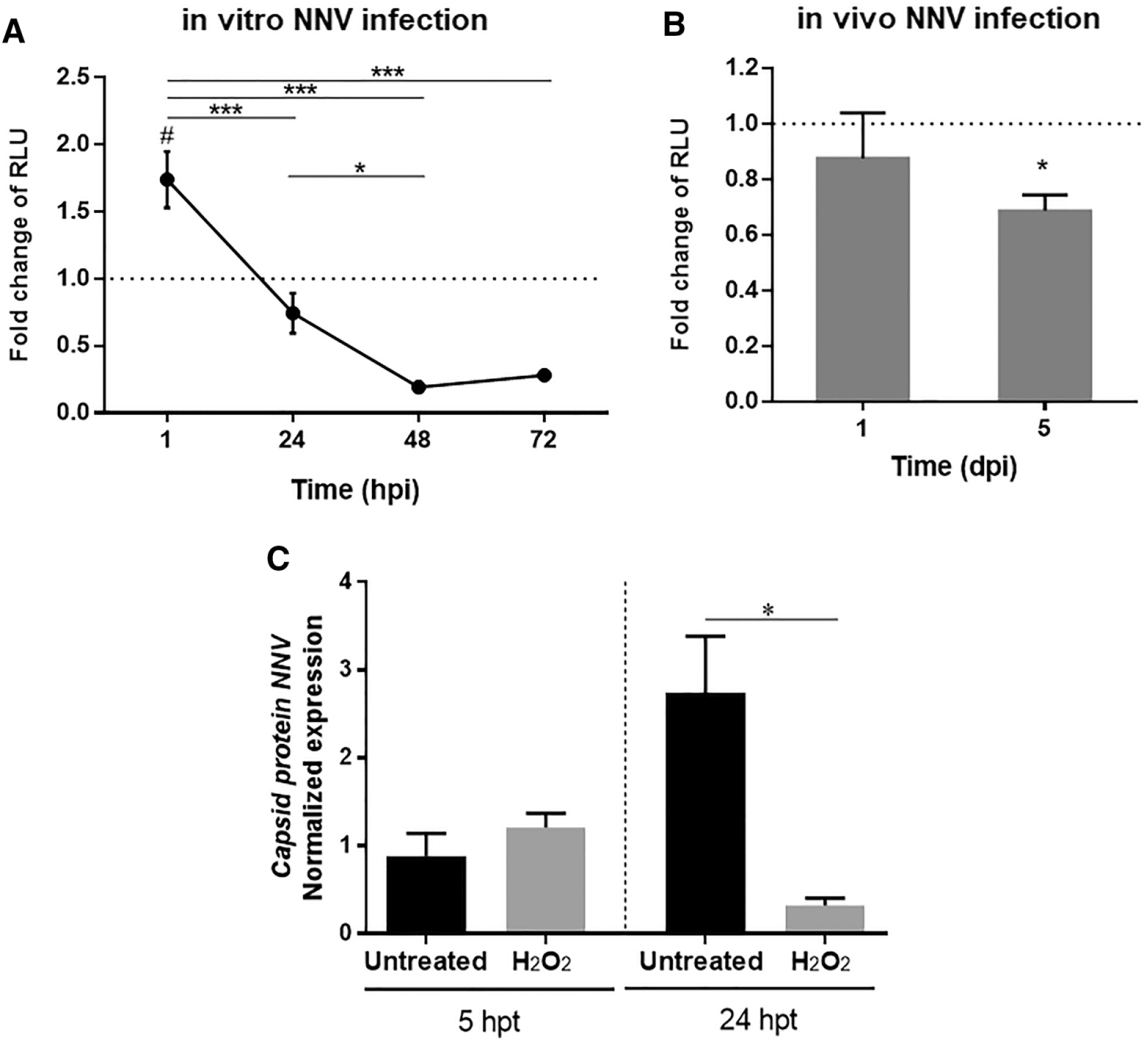
**Effect of NNV on cellular ROS production and the antiviral activity of oxygen radicals**. **A** ROS production by head kidney leukocytes during in vitro infection with NNV. The data are represented as the fold change relative luminescence units (RLUs) in the infected samples compared to those of the uninfected cells at each sampling point. Statistically significant differences between NNV-infected and control cells were represented with #, whereas differences along time for the infected cells were represented with *. **B** ROS detection in head kidney leukocytes at 1 and 5 dpi after in vivo infection with NNV. The data are represented as the fold change of the RLUs in the infected fish compared to those measured for the uninfected animals. **C** Replication levels of NNV after treatment with H_2_O_2_ measured by qPCR amplification of the gene encoding the NNV capsid. hpt: hours post-treatment.

Cortisol synthesis involves the production of ROS due to the activity of the steroidogenic cytochrome P450 enzymes (Quinn and Payne, 1984, 1985; Hornsby, 1987). However, at 72 hpi, we observed the downregulation of some genes encoding enzymes implicated in ROS production in the head kidney, including *ero1*-*like protein alpha* (*ero1a*), *hypoxia up*-*regulated protein 1* (*hyou1*), *protein disulfide*-*isomerase a4* (*pdia4*) and *quinone oxidoreductase pig3* (*qorx*). Therefore, we can assume that there was a shift in ROS generation in the head kidney from 24 to 72 hpi. To better understand the effect of NNV on the production of ROS, we conducted the in vitro infection of head kidney cells and measured ROS production at different times post-infection. The results showed that at 1 hpi, there was a significantly higher ROS level in the infected cells (Figure 7A). However, after 24 h, ROS production decreased in the infected cells, and this pattern was maintained at 48 and 72 hpi (Figure 7A). These data could be explained by the downregulation of *ero1* in the late stages of infection, as the product of this gene is the largest producer of H_2_O_2_ in the ER [30]. When we analysed the modulation of ROS production in vivo at 1 and 5 dpi, we found that ROS production in head kidney cells decreased with the time of infection, showing significant inhibition at 5 dpi compared to the uninfected fish (Figure 7B).

To investigate how the levels of ROS could affect the replication of NNV, we further evaluated the antiviral effects of these radicals by using H_2_O_2_. We found that when the virus was incubated with H_2_O_2_ for 5 h, there was no appreciable effect on NNV replication in SNN-1 cells (Figure 7C). However, when the treatment was maintained for 24 h, viral replication was significantly reduced compared to that of the non-treated virus (Figure 7C).

### Calcium modifications interfere with ROS production and nodavirus replication

**Figure 8:**
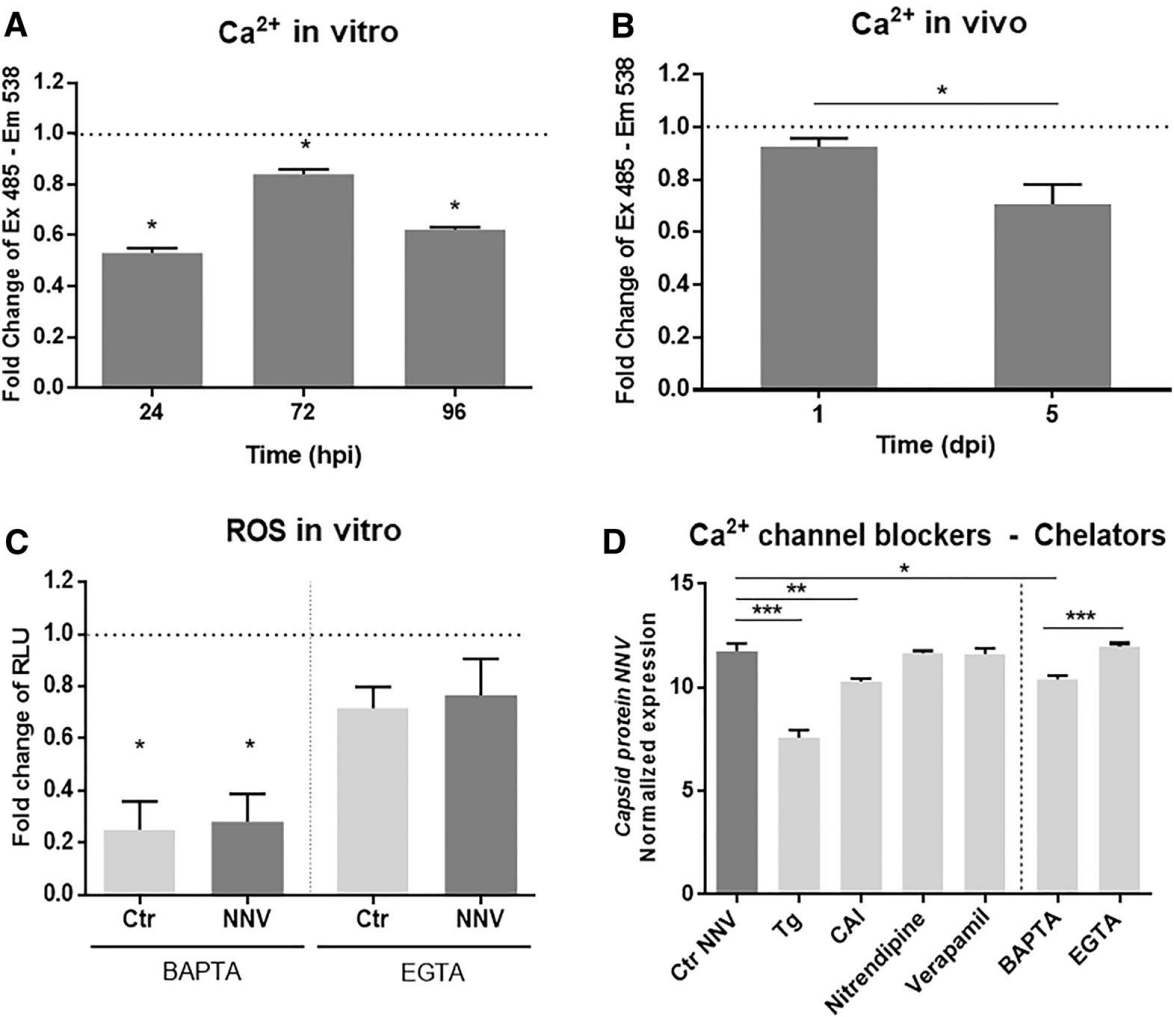
**Modulation of the intracellular calcium content by NNV infection and the effect of cytoplasmic calcium availability on ROS production and NNV replication. A** Intracellular calcium measurement with the fluorescent probe FLUO-4 AM in SSN-1 cells during infection with NNV. Values are the fold change of the fluorescence value obtained for the infected cells compared to the uninfected ones. **B** Calcium content measurement with FLUO-4 AM in brain cells isolated from in vivo NNV-infected sea bass and represented as the fold change value compared to the uninfected fish. **C** ROS production detection in head kidney leukocytes after in vitro pretreatment with the calcium chelators BAPTA-AM and EGTA and with or without NNV infection for 1 h. The data are represented as the fold change compared to untreated and uninfected cells. **D** Replication of NNV in SSN-1 cells after pretreatment with calcium channel blockers and chelators and infection with NNV for 48 h. Viral replication was detected by qPCR amplification of the gene encoding the NNV capsid.

We analysed the calcium load within SSN-1 cells during nodavirus infection by using the fluorescent probe FLUO-4 AM, which is used to measure calcium (Ca^2+^) concentrations inside living cells. As shown in Figure 8A, NNV-infected cells showed a lower Ca^2+^ concentration than uninfected cells at all tested times post-infection (24, 72 and 96 hpi). To further confirm the effect of the modulation of the genes encoding calcium transporters in the brain after NNV challenge, we also infected European sea bass in vivo; at 1 and 5 dpi, the brain was sampled, and the tissue was disintegrated to measure the Ca^2+^ concentration. As observed for the SNN-1 cells, the intracellular calcium concentration decreased with the time of infection (Figure 8B).

We further evaluated how these changes in the cellular calcium content in the infected cells could affect ROS production and NNV replication. For this purpose, we treated a primary culture of head kidney cells with BAPTA-AM or EGTA. We observed a significant decrease in ROS production when cells were treated with BAPTA-AM but not with EGTA, indicating that the sequestration of intracellular free calcium influences the production of ROS by cells (Figure 8C).

If infected cells show alterations in their calcium load and in the expression of different calcium channels (which in turn affect the production of ROS), the modulation of calcium could influence the replication of the virus. To better understand this effect, we tested different calcium channel blockers and calcium chelators. When SNN-1 cells were pretreated for 2 h with the channel blocker CAI, thapsigargin and the intracellular calcium chelator BAPTA-AM and then infected for 48 h, a significant reduction in the replication of NNV was observed compared to the untreated but infected cells (Figure 8D). These results demonstrate that although Ca^2+^ overload could negatively impact NNV replication, the virus requires Ca^2+^ availability.

### Validation of the RNA-Seq results

For the validation of the RNA-Seq results, some of the most differentially regulated genes in each tissue were selected and analysed by qPCR. For the validation of the brain data, the *b2 bradykinin receptor* (*bdkrb2*), *leukocyte cell*-*derived chemotaxin*-*2* (*lect2*), *prolactin (prl*), *fructose*-*bisphosphate aldolase* a (*aldoa*), *sacsin* (*sacs*) and *DNA damage*-*inducible transcript 4*-*like protein* (*ddit4l*) genes were chosen. For validation of the head kidney results, the *cholesterol side*-*chain cleavage enzyme mitochondrial* (*cyp11a1*), *cytochrome p450 11B mitochondrial* (*cyp11b1*), *steroid 17*-*alpha*-*hydroxylase/17,20 lyase* (*cyp17a1*), *steroid 21*-*hydroxylase* (*cyp21a*), *steroidogenic acute regulatory protein mitochondrial* (*star*) and *calreticulin* (*calr*) genes were chosen (Additional file 6). The Pearson’s correlation coefficient between the RNA-Seq and qPCR data was r = 0.903. Additionally, the expression of three genes significantly modulated in brain at 24 and 72 hpi (*lect2*, *prl* and *aldoa*) was analysed in an independent experiment composed by 5 individual biological replicates. For this experiment, the Pearson’s correlation coefficient between the RNA-Seq and qPCR data was r = 0.835 (Additional file 6).

## Discussion

Nervous necrosis virus (NNV) exhibits a neurotropic nature and causes damage to the nervous system (brain, retina and spinal cord) of infected fish [2]. For this reason, understanding how the brain responds to its first contact with the virus is critical for elucidating the antiviral strategies of this immune-privileged organ. The immune-privileged tissue concept suggests the existence of different conditions that help to control the access of pathogens to the central nervous system (CNS) but also the exacerbation of inflammation [31]. This is why both pro-inflammatory and anti-inflammatory cytokines are overexpressed after stimulation by several types of stressors, including pathogens and vaccines [32]. These responses are thought to be an evolutionary adaptation to protect indispensable organs with limited regeneration capacities from uncontrolled inflammation [33]. However, some pathogens, such as NNV, can overcome these physiological and immunological barriers and reach the CNS, but circulating immune cells can also migrate to the CNS and, together with resident immune cells, generate a response against the pathogen. A powerful immune response in the brain could lead to severe damage as a consequence of the activated pro-inflammatory mechanisms, whereas the absence of response could allow the persistence and spreading of the pathogen. This situation generates interesting an interplay between “fighting” and “tolerance”.

Here, we sought to study the response of *D. labrax* to infection by NNV, one of the most threatening pathogens in the culture of this important commercial fish species. Some previous studies have revealed the overexpression of certain immune genes after in vivo or in vitro infection with NNV in both European sea bass [14–22] and other fish species [5, 6, 8, 10–13, 20, 34], but the present study is the first to conduct RNA-Seq in *D. labrax* after in vivo infection with NNV.

Among the most differentially modulated genes in the brain, we observed high representation of pituitary hormones involved in the hypothalamic-pituitary-interrenal (HPI) axis, which is equivalent to the hypothalamic–pituitary–adrenal (HPA) axis of mammals. The HPI axis is activated under stress conditions and culminates in the secretion of cortisol by interrenal cells [35]. Cortisol is the main active corticosteroid in fish [36], and its secretion is induced by the release of adrenocorticotropic hormone (ACTH) by the pituitary gland. The released ACTH activates the steroidogenic signalling pathway, leading to cortisol secretion as the final product of HPI axis activation [35].

**Figure 9:**
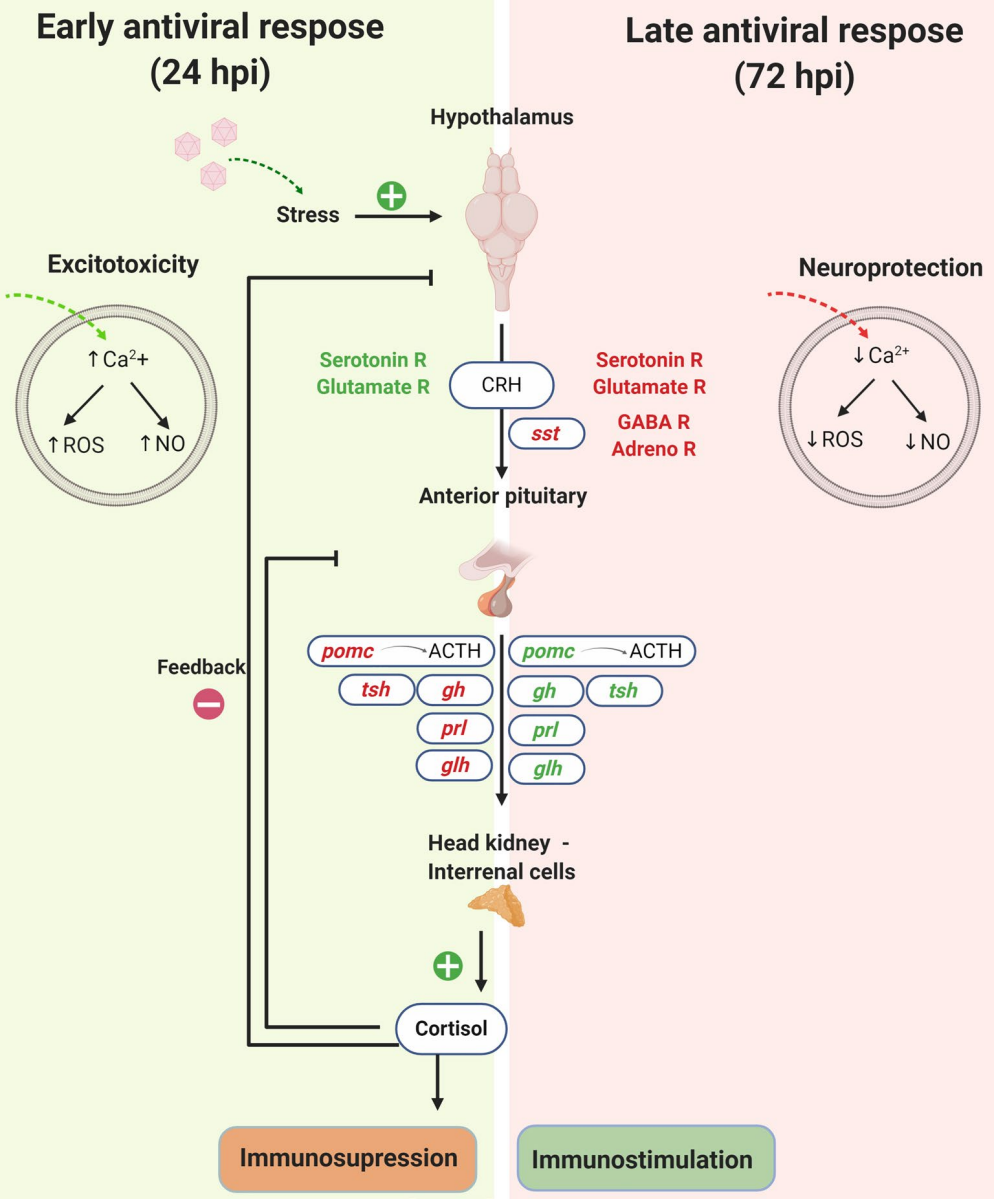
**Schematic representation of the predicted alterations of the hypothalamic-pituitary-interrenal (HPI) axis after NNV infection and its associated effects based on the transcriptome data.** Green indicates genes upregulated according to the RNA-Seq data; red indicates genes downregulated according to the RNA-Seq data.

Based on our transcriptome data, this stress response seemed to be activated at 24 hpi but attenuated after 72 h (Figure 9). At the earlier sampling point, we found overexpression of the serotonin and glutamate receptors in the brain, both of which are activators of the HPA axis by increasing corticotropin-releasing hormone (CRH) signalling systems [37, 38]. However, we did not observe changes in *crh* gene expression after NNV infection, and even the genes encoding pituitary hormones were downregulated at this point, including *pomc* and, consequently, ACTH levels. The only genes that were differentially modulated in the head kidney were those encoding enzymes involved in the synthesis of cortisol from cholesterol, which is indicative of HPI axis activation (Figure 9). Indeed, the downregulation of pituitary hormones could be explained by the negative feedback established between cortisol release and the recurrent synthesis of hypothalamic and pituitary hormones [35]. Similar results were observed in the rainbow trout head kidney after treatment with *Vibrio* bacterin [32]. Moreover, our results are supported by a recent paper on the effects of vaccine exposure in seabream (*Sparus aurata*). Thus, the gene expression responsiveness of the brain and pituitary to biotic stressors is low compared to the response induced by a physical stressor such as air exposure, meaning that acute abiotic stressors generate a significant neuroendocrine reaction, whereas biotic stressor reactions are modulated in the brain and pituitary [39].

The effect of stressors in animals involves a significant allostatic load, i.e., a sudden requirement for energy and resources. This means that most of the immediately available resources are diverted to stress response mechanisms to cope with the stressor, particularly those related to the initial rapid fight and flight responses (cardiovascular, respiratory, tissue perfusion; see [40]), while more routine metabolic and growth processes are downregulated. Therefore, responses that require a significant amount of energy and take some time, such as some immune responses, are delayed or postponed. This could explain the lack of response of immune genes observed at early time points (24 h) in the present work, as found for the Ig genes. When a stressor lasts longer, regulatory and feedback mechanisms allow other responses to the situation, as occurred after 72 h. This explains why genes related to growth and energetics, such as those encoding prolactin, somatolactin and somatotropin (Table 3), were severely downregulated at 24 h but upregulated at 72 h.

Cortisol modulates the immune response by provoking immunosuppression [23, 41]. Consequently, we observed the nearly total absence of an immune response at 24 hpi, and some immune-related genes were even downregulated at that time (including the gene encoding IgM), which could also be explained by high cortisol levels. In previous works based on qPCR analysis, overexpression of inflammation and antiviral immune genes was observed both in head kidney and brain from *D. labrax* infected with NNV [15, 17–19]. One of the most commonly analysed genes is the type I interferon-stimulated gene *myxovirus resistance gene* (*mx*) [15, 17–19], suggesting the activation of the main antiviral pathway, the type I interferon response. The absence of this response in our RNA-Seq results could be directly conditioned by the high stress response reached in the animals, which at the same time could influence the survival. Pijanowski et al. observed that those common carp (*Cyprinus carpio*) lines more susceptible to bacterial and parasite infection also showed a higher ability to respond to stress, whereas the highly resistant lines showed modest modulation of stress-related factors, revealing a certain potential correlation between stress response and infectious disease susceptibility [42]. Based on this, the genetic background of the animals could be conditioning both the stress and immune response.

It has been previously suggested that the adaptive immune response is critical in the defence against NNV, even at early times post-infection [22, 43–45]. Considering this suggestion and the observed overexpression of the genes involved in cortisol synthesis in the head kidney, we further investigated whether cortisol overload increases the susceptibility of European sea bass to NNV. However, although the administration of cortisol implants in the peritoneum increased the susceptibility of European sea bass to NNV, they did not negatively affect the synthesis of IgM. Although, in general terms, stressful conditions or cortisol administration decreased the IgM levels in different teleost species, some exceptions were observed (see [46]). Based on our results and the conclusions by Parra et al. [46], it seems that the regulation of the immune system by the neuroendocrine machinery is more complex than the interaction of cortisol with its receptors, and other components could be involved in the control of the immune response. Indeed, in the present work we observed that at 72 hpi higher IgM levels were found in those animals infected with NNV and carrying the cortisol implants compared to those infected but inoculated with the vehicle, suggesting that the synergy of the two stimuli may have increased IgM synthesis. High prolactin levels have been previously related to IgM synthesis in rainbow trout (*Oncorhynchus mykiss*) [47], and we confirmed that *prl* gene expression was also elevated at 72 hpi in the presence of the combination of the cortisol implant + NNV. How the combination of cortisol and NNV infection potentiates the expression of *prl* remains to be elucidated.

The hyperactivation of neurotransmitter receptors, especially glutamate receptors, generates excitotoxicity, which is defined as cell death resulting from the toxic effects of excitatory amino acids [29]. Prolonged exposure to glutamate generates an excessive influx of Ca^2+^ into neurons, which is highly neurotoxic and results in neuronal degeneration by increasing the production of nitric oxide (NO) and ROS, among other mechanisms [29]. Indeed, at 24 hpi, the expression of genes involved in calcium transport and cellular homeostasis seemed to be directed toward increasing Ca^2+^ levels into the cytoplasm, and some genes involved in ROS production, such as *ecto*-*NOX disulfide*-*thiol exchanger 1* (*enox1*), were also overexpressed (Figure 9). As a consequence, the host response at this early point could favour neuronal damage as well as an immunosuppressive status. The high ROS production associated with increased intracellular calcium would be harmful to NNV, as shown in the present work. Indeed, whereas physiological levels of intracellular Ca^2+^ are necessary for efficient NNV replication, an excess of Ca^2+^ would be pernicious. Calcium ions are required for the efficient virion assembly and infectivity of betanodaviruses [48]. As we also observed in this work, the NNV was able to modulate the ROS production, and this seems to be highly dependent on Ca^2+^ availability. However, ROS production tended to decrease with time. The transcriptome response also completely changed at 72 hpi, most likely as a response to protect neurons from the damage caused by excitotoxicity or to overcome the immunosuppressive effect of cortisol (Figure 9). The overexpression of serotonin and glutamate receptors practically disappeared, and some of these receptors were even downregulated at this sampling point. Moreover, the *immunoglobulin superfamily member 11* (*igsf11*) gene, encoding an adhesion molecule implicated in the synaptic stabilization of glutamate receptors [49], was inhibited, as was the *glucocorticoid modulatory element*-*binding protein 1* (*gmeb1*) gene, which produces a protein that increases sensitivity to glucocorticoids [50]. Interestingly, the most upregulated neurotransmitter receptor at 72 hpi was *GABA(A) receptor subunit beta*-*1* (*gbrb1*), which inhibits HPA axis activation [50]. Accordingly, three adrenergic receptors that mainly play an excitatory role in the regulation of the HPA axis [51] were downregulated. As expected, the modulation of cellular calcium regulators completely changed after 72 h, and most of the genes encoding Ca^2+^ importers were downregulated. Consequently, the genes encoding the enzymes involved in NO and ROS production were not induced, and even the nitric oxide synthase gene *nos1* was downregulated. It has been previously demonstrated that the inhibition of neuronal nitric oxide synthase protects against excitotoxicity [52].

The genes encoding the hypothalamic hormone *somatostatin* (*sst*) and *somatostatin receptor type 2* (*sstr2*) were inhibited at 72 hpi, which probably led to the overexpression of the pituitary hormone repertoire (Figure 9), due to its inhibitory activity in the pituitary gland [53]. Among the pituitary hormones, prolactin plays a critical stimulatory role in the proliferation of immune cells and the synthesis of immune factors [54] but also acts as a neuroprotective factor by reducing the Ca^2+^ overload induced by an excess of glutamate [55]. As a consequence, the production of NO is inhibited by prolactin [56]. Additionally, the *pomc* gene is overexpressed at this time point, based on which we might expect an upregulation of the genes involved in the synthesis of cortisol, which were not found to be significantly affected. At the same time, cortisol establishes negative feedback towards pituitary hormones, including ACTH, to control the magnitude of glucocorticoid release [35], which could explain the downregulation of *pomc* at 24 hpi but its higher expression after 72 h. As a consequence of all these modulations, the antiviral response might be more active at this time point, but although some relevant immune genes were overexpressed after 72 hpi, the response was still very discrete. At later sampling points, the antiviral response would most likely be more pronounced. Indeed, in the experiment involving cortisol implants, NNV induced the expression of *ighm* at 72 hpi, but this probably depends on the intensity of infection.

Alterations in the HPA axis have been observed in association with neurotropic and non-neurotropic virus and bacterial infections in mammals and even in association with viral and bacterial components [57, 58]. However, this is the first time that the modulation of the HPI axis has been described under teleost neurotropic virus infection. Although some of the processes described in this work need to be studied in a more detailed way, it seems that the initial boundless stress response is counteracted by the host. This could be a strategy to reduce the neural damage associated with excitotoxicity and a mechanism for increasing the immune response. Nevertheless, a higher immune response would increase inflammation due to the activity of pro-inflammatory cytokines, which could be the reason for the high susceptibility of European sea bass to NNV: an equilibrium between the neuroprotective environment and efficient immune response is difficult to achieve. The study of this response in fish species resistant to NNV, such as gilthead seabream (*Sparus aurata*) [59], could help to better elucidate this complex interplay.

## Supplementary information

**Additional file 1.** **Sequence of the primer pairs used in this experiment.**

**Additional file 2. Video showing the erratic swimming behaviours of juvenile European sea bass infected with NNV.**

**Additional file 3. Differential expression analysis in the brain at 24 and 72 hpi with NNV.**

**Additional file 4. Differential expression analysis in the head kidney at 24 and 72 hpi with NNV.**

**Additional file 5. Schematic representation of the contigs significantly differentially modulated in the head kidney at 24 hpi with NNV.** These DEGs mainly consisted of the genes encoding those enzymes involved in the last steps of cortisol synthesis.

**Additional file 6. Validation of the RNA-Seq results by qPCR.** A) Comparison of RNA-Seq and qPCR data for genes significantly modulated in brain. B) Comparison of RNA-Seq and qPCR data for genes significantly modulated in head kidney. C) Correlation between the RNA-Seq and qPCR data. D) Validation of three genes significantly modulated in brain at 24 and 72 hpi in an independent experiment.

## Data Availability

The read sequences were deposited in the NCBI Sequence Read Archive (SRA) under accession number PRJNA589774.

## Abbreviations

BSA: Bovine serum albumin
DEGs: differentially expressed genes
DMSO: dimethyl sulfoxide
Dpi: days post-infection
ELISA: enzyme-linked immunosorbent assay
FBS: foetal bovine serum
FDR: false discovery rate
GO: Gene ontology
HPA: Hypothalamic–pituitary–adrenal
Hpi: hours post-infection
HPI: hypothalamic-pituitary-interrenal
i.m.: intramuscularly
i.p.: intraperinoteally
NNV: nervous necrosis virus
PBS: phosphate-buffered saline
PMA: phorbol myristate acetate
RGNNV: red-spotted grouper nervous necrosis virus
RNA-Seq: RNA sequencing
ROS: reactive oxygen species
RT: room temperature
TPM: transcripts per million
VER: viral encephalopathy and retinopathy
