# Supplementary figures and images for: RNA-Seq analysis of European sea bass (Dicentrarchus labrax L.) infected with nodavirus reveals powerful modulation of the stress response
Source: Vet Res. 2020 May 12;51:64. doi: 10.1186/s13567-020-00784-y (PMC7218500; doi:10.1186/s13567-020-00784-y)

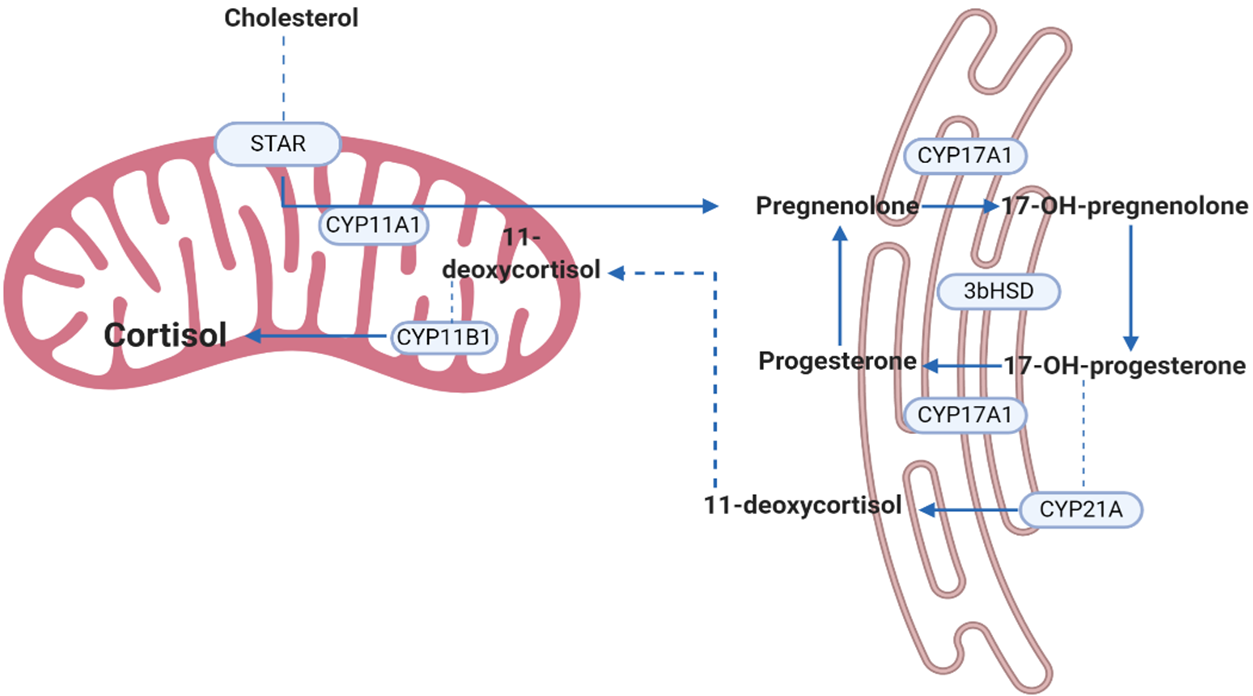

Supplement: Supplementary file 5 — Additional file 5. Schematic representation of the contigs significantly differentially modulated in the head kidney at 24 hpi with NNV. These DEGs mainly consisted of the genes encoding those enzymes involved in the last steps of cortisol synthesis. [file 13567_2020_784_MOESM5_ESM.tif]

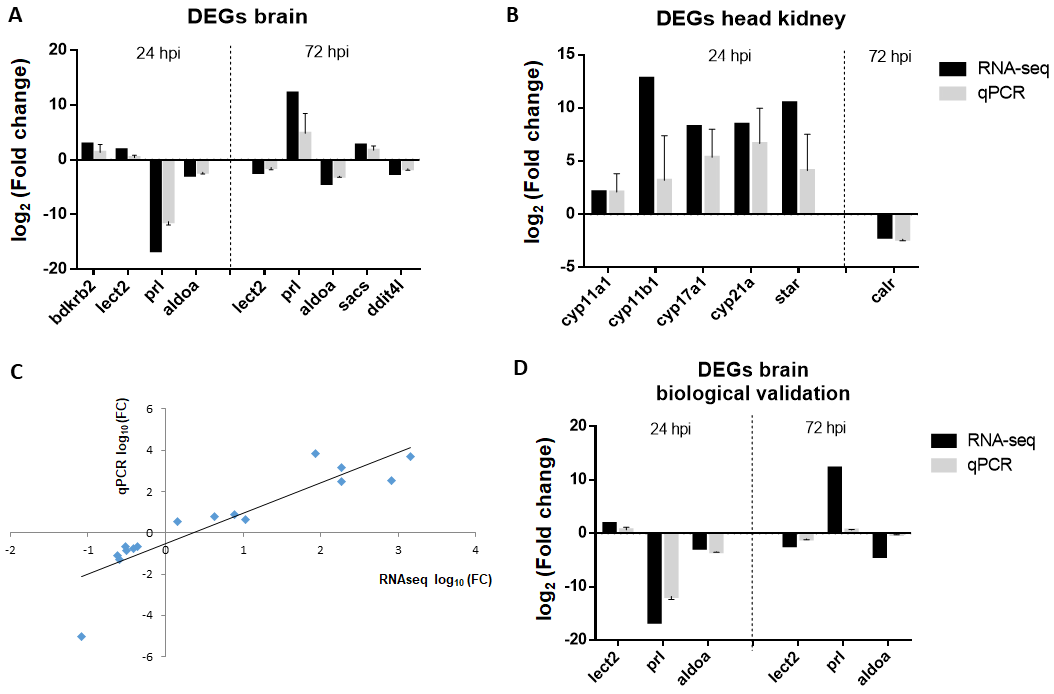

Supplement: Supplementary file 6 — Additional file 6. Validation of the RNA-Seq results by qPCR. A) Comparison of RNA-Seq and qPCR data for genes significantly modulated in brain. B) Comparison of RNA-Seq and qPCR data for genes significantly modulated in head kidney. C) Correlation between the RNA-Seq and qPCR data. D) Validation of three genes significantly modulated in brain at 24 and 72 hpi in an independent experiment. [file 13567_2020_784_MOESM6_ESM.tif]
